# Identification of 8-Azaguanine Biosynthesis–Related Genes Provides Insight Into the Enzymatic and Non-enzymatic Biosynthetic Pathway for 1,2,3-Triazole

**DOI:** 10.3389/fbioe.2020.603514

**Published:** 2020-11-05

**Authors:** Feifei Hou, Yupeng Wan, Qi Gan, Mo Xian, Wei Huang

**Affiliations:** ^1^CAS Key Lab of Biobased Materials, Qingdao Institute of Bioenergy and Bioprocess Technology, Chinese Academy of Sciences, Qingdao, China; ^2^Department of Chemical Engineering, University of Chinese Academy of Sciences, Beijing, China

**Keywords:** 1, 2, 3-triazole, biosynthetic genes, nitric Oxide synthase, metabolites, *in vitro* and feeding experiments

## Abstract

8-Azaguanine (1) is a special 1,2,3-triazole containing natural product that possesses potent antibacterial and antitumor activities. In the present study, the entire 8-azaguanine biosynthetic gene cluster was located from *Streptomyces* CGMCC4.1633. Targeted gene disruption, heterologous expression analysis, and feeding experiments identified crucial genes for 8-azaguanine production. Moreover, we characterized the structure of two novel metabolites, analyzed NO (or reactive nitrogen species) related genes 8-azgA/B and radical SAM enzyme homologous 8-AzgG, and verified the non-enzymatic ring formation reaction of 8-azaguanine 1,2,3-triazole. All of the data and presumptions provide insight into the timing and mechanism of the enzymatic and non-enzymatic pathway that produce 8-azaguanine-type 1,2,3-triazole.

## Graphical Abstract

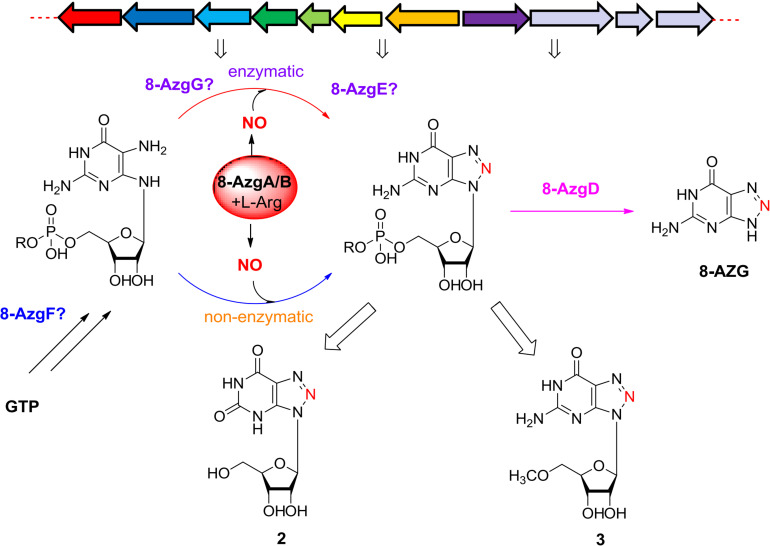

## Introduction

**FIGURE 1:**
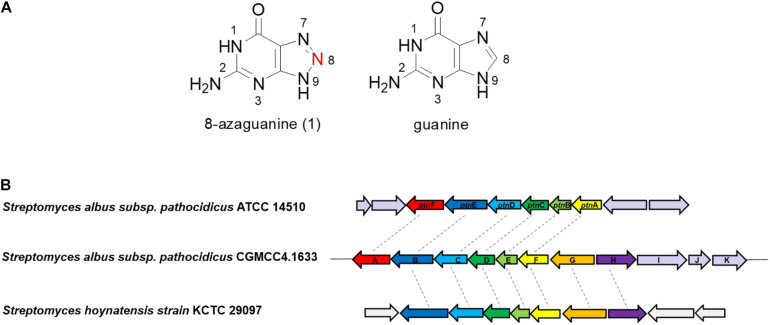
**(A)** The structures of 8-azaguanine (1) and guanine. **(B)** Organizations of the 8-*azg* biosynthetic gene clusters and the comparison with *ptn* gene cluster.

8-Azaguanine (8-AZG, 1, also known as pathocidin) is a triazole analog of guanine (Figure 1A), which was first found in *Streptomyces albus* var. *pathocidicus* culture medium as a secondary metabolite with activities including antibacterial and antitumor (Anzai et al., 1961), such as polyploid-specific effects to high-ploidy breast cancer (Choudhary et al., 2016); potential activity against *Mycobacterium tuberculosis* (Sao Emani et al., 2018); modulating HIF levels in neoplastic cells (Xia et al., 2009); stabilizing mutated von Hippel–Lindau protein (Ding et al., 2012); and direct potentiation of natural killer cell cytotoxicity (Kim et al., 2019). It competes with guanine to disturb the modification of tRNA and interferes with the initiation of translation by suppressing the formation of 43S and 80S ribosome initiation complexes to inhibit protein synthesis. Structural analysis showed that 8-AZG possesses a rare three-consecutive-nitrogen-atom triazole, which has only been discovered in 8-AZG in natural products. In recent years, natural products N-N bond formation studies had aroused great interest among the researchers, for example, the diazo moiety of cremeomycin and kinamycin; construction of N-nitrosating part in streptozotocin; azoxy bond formation for azoxymycins; biosynthesis of piperazate for kutzneride; hydrazine-forming machinery in s56-p; and N-hydroxytriazenes moiety for triacsins (Waldman et al., 2015; Sugai et al., 2016; Du et al., 2017; Matsuda et al., 2018; Wang et al., 2018; Guo et al., 2019; He et al., 2019; Ng et al., 2019; Twigg et al., 2019). Therefore, the unique 1,2,3-triazole structure and potent biological activities of 8-AZG have attracted our special attention.

Although earlier works in the feeding experiments with ^14^C-labeled compounds demonstrate [2-^14^C] guanine could be efficiently incorporated into 8-AZG, [8-^14^C] guanine was not incorporated (Hirasawa and Isono, 1978). According to these results, we presume that the skeleton of 8-AZG is formed from guanine or its derivatives by the replacement of C-8 with unknown source of nitrogen (Figure 1A). The pathway responsible for 8-AZG formation and the enzymatic logic for triazole biosynthesis have not yet been elucidated in detail. We noticed the study of another group (Zhao et al., 2020), completed at the same time as our work. They have reported the biosynthetic gene cluster mining for 8-AZG and carried out a series of *in vivo* and thorough *in vitro* work to reveal the biosynthetic route to the natural product 8-AZG. Here we present the identification of the 8-AZG biosynthetic gene cluster (BGC) from *S. albus pathocidicus* CGMCC4.1633 through genome scanning. Targeted gene disruption and heterologous expression revealed important genes related to 8-AZG biosynthesis. Functional assignments were made for each gene by comparing the deduced amino acid sequence with proteins of known function. Feeding experiments, mutant intermediates structural elucidation, and *in vitro* tests explore the biosynthetic pathway for 8-AZG. These two independent studies drew similar conclusions and complemented the results of each other’s works.

## Materials and Methods

### General Experimental Procedures

Liquid chromatography high-resolution mass spectrometry (LC-HRMS) analysis was performed on an Agilent Technologies 6520 Accurate-Mass Q-TOF LC-MS instrument with an Agilent Eclipse Plus C18 column (4.6 × 100 mm). A linear gradient of 2–95% CH_3_CN (vol/vol) over 20 min in H_2_O with 0.1% formic acid (vol/vol) at a flow rate of 0.2 mL/min was used. High-performance liquid chromatography (HPLC) analysis was carried out with Nexera XR HPLC apparatus using an Agilent 5 TC-C18 (2) (250 × 4.6 mm). Nuclear magnetic resonance (NMR) spectra were recorded on a Bruker Biospin 600 MHz spectrometer. Polymerase chain reaction (PCR) primers were synthesized by Tsingke Biological Technology. Kits for plasmid preparation and DNA extraction were from Omega. Standard molecular biology methods were used for all other DNA manipulations. *N*^G^-hydroxyl-L-arginine was purchased from Sigma. 8-AZG was purchased from Aladdin.

### Genome Sequencing and Analysis

The *S. albus* subsp. *pathocidicus* CGMCC4.1633 genome was sequenced using Illumina technology. The DNA sequence of the 8-*azg* gene cluster has been deposited into GenBank under the accession number no. MT543149. Analysis of the open reading frames (ORFs) and function predictions were carried out by Frame Plot 4.0 beta^1^, NCBI BLAST^2^.

### Construction of Gene Inactivation Mutants in *S. albus* subsp. *pathocidicus* CGMCC4.1633

*In vivo* generation of targeted mutations in *S. albus* subsp. *pathocidicus* CGMCC4.1633 was achieved by conjugative transfer of disruption plasmids from *Escherichia coli* S17-1 to *S. albus* subsp. *pathocidicus* CGMCC4.1633 according to standard protocols. A *Hin*dIII/*Xba*I fragment and an *Xba*I/*Eco*RI fragment flanking the gene of interest were successively ligated and cloned into the *Hin*dIII/*Eco*RI sites of pKC1139 to yield a knockout cassette. The resulting plasmids were then introduced to replace the genes of interest (8-*azgA-K*, 8-*azgA*, 8-*azgB*, 8-*azgC*, 8-*azgD*, 8-*azgE*, 8-*azgF*, 8-*azgG*, 8-*azgH*, 8-*azgI*, 8-*azgJ*, 8-*azgK*) using homologous recombination and were transformed into *E. coli* S17-1 for conjugation with *S. albus* subsp. *pathocidicus* CGMCC4.1633. The apramycin-resistant (50 μg/mL) colonies were identified as single-crossover mutants. The single-crossover mutants were further incubated without antibiotics and then screened for apramycin-sensitive clones. Among these clones, the double crossover mutant was selected and verified by PCR (Supplementary Figure S1). Mutant analysis was carried out following the procedures detailed below. LC-HRMS analysis was performed on an Agilent Technologies 6520 Accurate-Mass Q-TOF LC-MS instrument with an Agilent Eclipse Plus C18 column (4.6 × 100 mm). A linear gradient of 2 to 95% CH_3_CN (vol/vol) over 20 min in H_2_O with 0.1% formic acid (vol/vol) at a flow rate of 0.2 mL/min was used. HPLC analysis was carried out with Nexera XR HPLC apparatus using an Agilent 5 TC-C18 (2) (250 × 4.6 mm). Elution was performed at 1 mL min^–1^ with a mobile-phase mixture consisting of a linear gradient of water and acetonitrile [(vol/vol): 98:2, 0–10 min; 90:10, 10–15 min; 80:20, 15–20 min; 98:2, 20–25 min], both of which contain 0.1% (vol/vol) trifluoroacetic acid.

### Cloning, Overexpression, and Purification of Proteins

The gene 8-azgA was PCR amplified using the primers listed in Supplementary Table S2 and *S. albus* subsp. *pathocidicus* CGMCC4.1633 genomic DNA as the template. Purified PCR products were ligated to pET-duet (Novagen) following standard protocols and confirmed by DNA sequencing. The resulting expression constructs were transformed into *E. coli* BL21 (DE3)-pGro7 cells. Expression and purification for all hexahistidine-tagged proteins followed the general procedure and are detailed as follows: in 1 L of liquid culture, the cells were grown at 37°C in LB medium with 100 μg/mL ampicillin and 50 μg/mL chloramphenicol until an OD600 of 0.4. The cells were cooled on ice for 10 min and then induced with 0.1 mM isopropyl-β-D-1-thiogalactopyranoside and 0.5 mg/mL L-arabinose for 16 h at 16°C. The cells were harvested by centrifugation (5,000 *g*, 15 min, 4°C), resuspended in 30 mL lysis buffer (25 mM HEPES, pH 7.5, 0.5 M NaCl, 5 mM imidazole) and lysed by homogenization on ice. Cellular debris was removed by centrifugation (13,000 *g*, 1 h, 4°C). The soluble proteins were incubated with 0.5 mL of Ni-NTA agarose resin (Qiagen) at 4°C for 1 h and loaded onto a gravity flow column. The proteins were washed with washing buffer (20 mM HEPES, pH 7.5, 300 mM NaCl, and 20 mM imidazole) and eluted with elution solution (20 mM HEPES, pH 7.5, 300 mM NaCl, and 150 mM imidazole). Purified proteins were concentrated and buffer exchanged into protein stock buffer (20 mM HEPES, pH 7.5, 300 mM NaCl, and 10% glycerol) using Amicon Ultra filters. The final proteins were flash-frozen in liquid nitrogen and stored at −80°C.

### *In vitro* Biochemical Assays Using 8-AZG Biosynthetic Enzymes

For the *in vitro* assay of 8-AzgA, the reaction mixture (50 μL) contained 8-AzgA or GroEL (chaperone protein expressed by pGro7)^1^, 0.5 mM *N*^G^-hydroxyl-L-arginine (Sigma–Aldrich), and H_2_O_2_ (40 mM) in H_2_O (pH 6.8). The reaction mixture was incubated for 1 h at room temperature, followed by nitrite detection using Griess reagent I and II. Chaperone was used as a negative control. At least three independent replicates were performed for each assay, and representative results are shown.

### *In vitro* Non-enzymatic Triazole Formation

For the *in vitro* non-enzymatic reactions of compound 5 (4-hydroxy-2,5,6-triaminopyrimidine) with the nitric oxide donor diethylamine (DEA) NONOate or sodium nitrite, reaction mixtures (100 μL) contained 0.5 mM 5 and 1 mM DEA NONOate or 10 mM sodium nitrite in H_2_O (pH 6.8). The reaction mixture was incubated for 1 h at room temperature before HPLC analysis.

### Heterologous Production and Feeding Experiments in *S. albus* J1074

For *in vivo* reconstitution, different combinations of 8-*azg* genes were cloned and arranged in the vector pSET152. After confirmation with restriction and sequencing analysis, these plasmids were introduced into heterologous *S. albus* J1074 strains by conjugation from *E. coli* S17-1 to give corresponding engineered *S. albus* J1074 strains according to the standard procedure.

For the feeding experiments, NaNO_2_ was added at final concentrations of 10 mM at 48 h. After another 48 h, cultures were centrifuged at 13,000 revolutions/min (rpm) for 5 min to remove the mycelium and subjected to filtration before HPLC or LC-MS analysis.

### Small-Scale Production and Isolation of 8-AZG Biosynthetic Intermediates

To screen for 8-AZG production, starter cultures in TSB medium were inoculated with mycelia and incubated at 30°C, 200 rpm for 48 h; 1 mL of the starter culture was then inoculated into modified medium [(g L^–1^): soybean ice powder (20), glycerol (20), beef extract powder (3), NaCl (3)], and the cultures were grown at 30°C, 200 rpm for 4 days. 8-AZG and its biosynthetic intermediates supernatants were then subjected to filtration before HPLC or LC-MS analysis. Analytic HPLC analysis was carried out with Nexera XR HPLC apparatus using an Agilent 5 TC-C18 (2) (250 × 4.6 mm). Elution was performed at 1 mL min^–1^ with a mobile-phase mixture consisting of a linear gradient of water and acetonitrile (vol/vol: 98:2, 0–10 min; 90:10, 10–15 min; 80:20,15–20 min; 98:2, 20–25 min), both of which contain 0.1% (vol/vol) trifluoroacetic acid. Authentic compound of 8-AZG (Aladdin) was used as a control (detection wavelength: 245 nm). LC-HRMS analysis was performed on an Agilent Technologies 6520 Accurate-Mass Q-TOF LC-MS instrument. A linear gradient of 2 to 95% CH_3_CN (vol/vol) over 20 min in H_2_O with 0.1% formic acid (vol/vol) at a flow rate of 0.2 mL/min was used. Large-scale production and isolation of the biosynthetic intermediates for structural analysis are described below.

### Large-Scale Production, Purification, and Characterization of 2 and 3

For large-scale production, 5 mL starter cultures of *S. albus* subsp. *pathocidicus* CGMCC4.1633 Δ8-*azgD* in TSB medium were inoculated with mycelia and incubated at 30°C, 200 rpm, for 48 h. One milliliter of the seed culture was then coated on solid plate [ISP2 agar (g L^–1^): malt extract (10), yeast extract (4), glucose (4), agar (20)] for static fermentation at 30°C for 5 days. 8-AZG biosynthetic intermediates were extracted from methanol. The solvent was removed by rotary evaporation, and the residue was redissolved in methanol (2 mL). Compounds 2 and 3 from *S. albus* subsp. *pathocidicus* CGMCC4.1633 Δ8*-azgD* were purified by Nexera XR HPLC apparatus using an Agilent 5 TC-C18 (2) (250 × 4.6 mm). Elution was performed at 1 mL min^–1^ with a mobile-phase mixture consisting of a linear gradient of water and acetonitrile (vol/vol: 98:2, 0–10 min; 90:10, 10–15 min; 80:20, 15–20 min; 98:2, 20–25 min), both of which contain 0.1% (vol/vol) trifluoroacetic acid. Fractions containing 8-AZG biosynthetic intermediates were collected manually and concentrated under vacuum. The resulting purified compounds 2 and 3 were dried and analyzed by HRMS and NMR. LC-HRMS analysis was performed on an Agilent Technologies 6520 Accurate-Mass QTOF LC-MS instrument with an Agilent Eclipse Plus C18 column (4.6 × 100 mm). A linear gradient of 2 to 95% CH_3_CN (vol/vol) over 25 min in H_2_O with 0.1% formic acid (vol/vol) at a flow rate of 0.2 mL/min was used. NMR spectra were recorded on a Bruker Biospin 600 MHz spectrometer.

*8-Azaxanthosine (2):* White amorphous powder; [α] – 47.2 (*c* 0.09, MeOH); UV (CH_3_CN) λ_max_ 254.7 nm; IR (KBr) ν_max_ 3,358, 2,930, 1,694, 1,629, 1,576, 1,404, 1,276, 1,055, 960, 804, 786, and 713 cm^–1^; ^1^H and ^13^C NMR data (Supplementary Table S4); HRESIMS *m/z* 286.0783 [M + H]^+^ (calcd for C_9_H_12_N_5_O_6_, 286.0782).

*8-azaguanosine (3):* White amorphous powder; [α] – 50.1 (*c* 0.10, MeOH); UV (CH_3_CN) λ_max_;205.1, 255.9 nmIR (KBr) ν_max_ 3,288, 2,933, 1,699, 1,620, 1,576, 1,362, 1,291, 1,092, 938, 801, 780, and 744 cm^–1^; ^1^H and ^13^C NMR data (Supplementary Table S4); HRESIMS *m/z* 299.1094 [M + H]^+^ (calcd for C_10_H_15_N_6_O_6_, 299.1098).

## Results

### Sequencing of *S. albus pathocidicus* CGMCC4.1633 and 8-AZG Biosynthetic Gene Cluster Analysis

**FIGURE 2:**
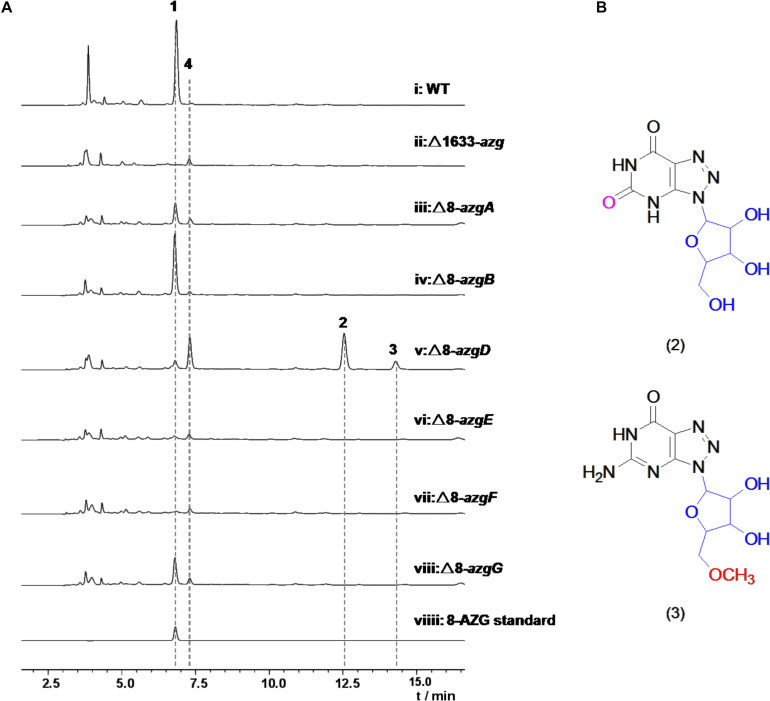
**(A)** HPLC analysis with UV detection (245 nm) of *Streptomyces albus* subsp. *pathocidicus* CGMCC4.1633 wild-type strain and the mutants. **(B)** Structures of metabolites 2 and 3. Compounds 2, 3 were isolated from mutant strain Δ8-*azgD*.

To identify the potential 8-AZG biosynthetic genes, the genome of *S. albus pathocidicus* CGMCC4.1633 was sequenced. The final genome assembly resulted in ∼5.9 M base pairs. Genome mining for 8-AZG BGC candidates was performed using a local BLASTP analysis on the genome with a GTP cyclohydrolase (GCH) probe, which might be involved in the first step hydrolytic cleavage of guanine moiety C-8 (Hirasawa and Isono, 1978). Through our bioinformatics search, one locus with clustered genes in which a gene encoding GCH I–like protein was located. Targeted gene disruption strain Δ1633-*azg* deleted 11-kb multigene region upstream and downstream of this area completely abolished the production of 8-AZG, confirming that the identified cluster is involved in the biosynthesis of 8-AZG (Figure 2A).

The putative 8-AZG BGC spans 11.5 kb and consists of 11 ORFs, here assigned as 8-*azgA-K*. In the article of Zhao et al., the *ptn* gene cluster contains six ORFs *ptnA-F*, which correspondingly in our gene cluster are 8-*azgF-A*. Among them, *ptnA*/8-*azgF* is a putative GCH I gene; *ptnB*/8-*azgE* encodes a hypothetical protein; PtnC/8-AzgD is a monophosphate nucleosidase; PtnD/8-AzgC is a didomain protein; PtnE/8-AzgB is a purine efflux pump protein; and PtnF/8-AzgA is a putative bacterial nitric oxide synthase (NOS) (Zhao et al., 2020). We also noticed the published genome of *Streptomyces hoynatensis* KCTC 29069 containing highly homologous genes to the 8-*azg* cluster (from 8-*azgB* to 8-*azgH*), and there are a few other secondary metabolic biosynthesis related genes nearby this region (Figure 1B), although no 8-AZG-type product has been reported from this strain.

### 8-AZG Biosynthetic Genes Disruption and Metabolite Characterizations

To further explore the functions of 8-*azg* ORFs, we disrupted every gene in the 8-*azgA-K* cluster by clean gene-knockout experiments. All of the genes were deleted in-frame through double crossover leaving a scar of 6 bp. 8-AZG production was completely abolished in Δ8-*azgF*, and no new metabolites were identified from this mutant culture by HPLC analysis (Figure 2A), demonstrating that GCH I homolog gene 8-*azgF* is crucial for the biosynthesis of 8-AZG. According to the reported activity of GCH I, the reaction catalyzed by 8-AzgF is supposed to involve the hydrolytic opening of GTP imidazole ring and then the removal of the C-8 carbon atom as formate affording the triaminopyrimidine derivative (Rebelo et al., 2003). The solubility of 8-AzgF is poor when expressed in *E. coli*; although we synthesized the homology gene of 8-*azgF* from *S. hoynatensis* KCTC 29069 and obtained some soluble protein, no reaction with GTP, GDP, GMP, or guanine was detected after several attempts.

Through testing, only trace amount of 8-AZG exists in the Δ8-*azgD*, Δ8-*azgE* mutants broth, which means 8-*azgD* and 8-*azgE* are important for 8-AZG production in *S. pathocidicus*. Two unidentified metabolites 2 and 3, which were not found in the wild-type or any other mutant cultures significantly accumulated in Δ8-*azgD* strain (Figure 2A). Then 2 and 3 were isolated from a scale-up fermentation culture (10 L) and structurally characterized by HRMS and NMR analysis, further assisted by comparison with the reported NMR data of known 8-AZGs (Supplementary Table S4 and Supplementary Figures 7–18). It indicated that compound 2 is 8-azaxanthosine, 8-azaxanthine with a ribose at N9, and 3 was identified as 8-AZG ribose (8-azaguanosine) congener, bearing a methoxyl group on the C5′ of 8-azaguanosine nucleoside (Figure 2B). Another metabolite, 8-azaguanosine (8-AZG-3) molecular weight (284) was only detected in Δ8-*azgD* mutant by HRMS (Supplementary Figures 19, 20). In addition, the yield of metabolite 4 was measurably higher in Δ8-*azgD* than the wild type and other mutant strains. According to the molecular weight detected by HRMS, we speculate that 4 is primary metabolite adenosine, and the comparison of 4 with authentic compound confirmed our inference (Supplementary Figure 6). Structurally, both nucleoside metabolites 2 and 3 possess ribose and triazole unit, which indicated that 1,2,3-triazole part of 8-AZG was constructed prior to ribose hydrolysis.

### Heterologous Expression of 8-AZG Genes

**FIGURE 3:**
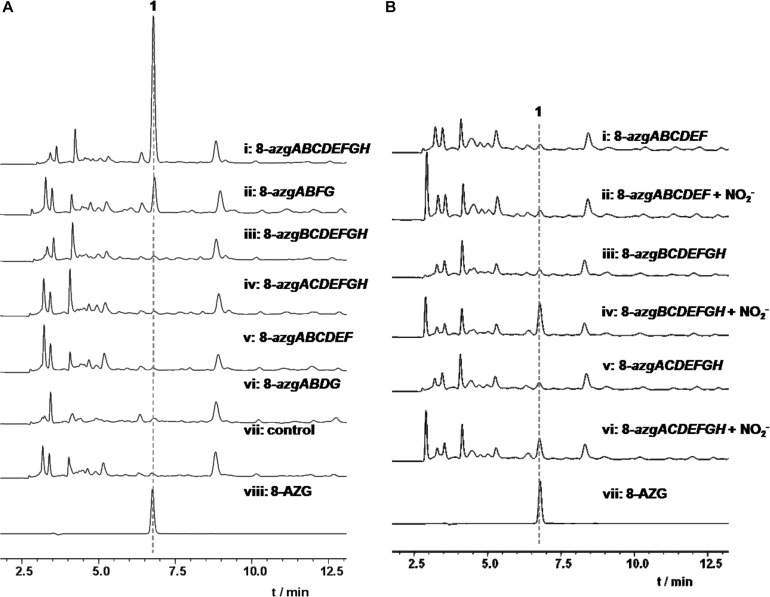
**(A)** HPLC analysis of 1 heterologous production in *S. albus* J1074. **(B)** HPLC analysis of 1 from NO${}_{2}^{-}$ (10 mM) feeding experiments in S. *albus* J1074 heterologous expression strains.

The titer of 8-AZG correspondingly decreased in Δ8-*azgA*, Δ8-*azgB*, Δ8-*azgC*, Δ8-*azgG*, Δ8-*azgH*, and Δ8-*azgJ* fermentation broth compared to the wild-type strain (Figure 2A and Supplementary Figure 4). This suggests that either these genes are not crucial for 8-AZG generation, or their functions are replenished by other genes outside the 8-*azg* cluster or some unknown factors. In order to verify the biosynthetic function of these genes, various combinations of 8-*azg* ORFs were cloned into pSET152 for heterologous expression in *S. albus* J1074. HPLC analysis of the culture extracts indicated that 8-*azgABCDEFGH* generated the maximum titer of 8-AZG, almost two-thirds of the native producer *S. pathocidicus*. A minimum set of four genes operon, 8-*azgABFG*, is able to produce a certain amount of 8-AZG in *S. albus* J1074 (Figure 3A). In 8-*azgABF*, 8-*azgABCDEF*, 8-*azgACDEFGH*, and 8-*azgBCDEFGH* heterologous expression strains, only a slight amount of 8-AZG was detected by LC-HRMS analysis (Supplementary Figure 3). The gene knockout and heterologous expression data exhibit that 8-*azgA/B/D/E* (or 8-*azgG*) genes are critical in 8-AZG biosynthetic pathway; however, their roles are compensated by the equivalents in *S. pathocidicus* and *S. albus* J1074. The disruption of 8-*azgI* had no effect on 8-AZG production. Δ8-*azgK* resulted in a small titer increase, so 8-*azgK* was considered to be a negative transcriptional regulator gene (Supplementary Figure 4).

### *In vitro* and Feeding Experiments

Bioinformatics analysis revealed that 8-*azgA* probably encodes an NOS being responsible for the oxidation of L-arginine (Arg) to release nitric oxide (Kers et al., 2004). Nitric oxide can be autoxidized and give different “reactive nitrogen species” products, such as nitrogen dioxide (NO_2_), dinitrogen trioxide (N_2_O_3_, which can also be formed from NO${}_{2}^{-}$ at acidic pH), etc. (Möller et al., 2019). To confirm the function of 8-AzgA, we purified its soluble protein; further enzymatic assays with *N*^G^-hydroxyl-L-arginine (intermediate of L-arginine and NOS reaction) and 8-AzgA revealed the release of NO in the reaction system (Supplementary Figure 2). Furthermore, sodium nitrite (10 mM) was separately fed into the 8-*azgABF*, 8-*azgBCDEFGH*, 8-*azgCDEFGH*, 8-*azgACDEFGH*, 8-*azgABDFG*, and 8-*azgABCDEF* containing *S. albus* J1074 strains. The titer of 8-AZG is obviously increased compared with no feeding control group except for 8-*azgABF*, 8-*azgABDFG*, and 8-*azgABCDEF* (Supplementary Figure 5). These results showed that the role of 8-AzgA and 8-AzgB could be replaced by feeding NO${}_{2}^{-}$ into the broth (Figure 3B). 8-AzgB has homology to MFS (major facilitator superfamily) transporter that is proposed to act as either nitrate/proton symporter or nitrate/nitrite antiporter (Goddard et al., 2008). But an attempt to overexpress 8-AzgB in *E. coli* was unsuccessful. It was thus hypothesized that there is an intriguing reactive nitrogen species generation or transportation system in 1,2,3-triazole biosynthetic pathway: first, NO is produced by the catalysis of NOS 8-AzgA; afterward, NO converts into N_2_O_3_ or some other reactive nitrogen species, and 8-AzgB is in charge of their transformation or transportation.

**FIGURE 4:**
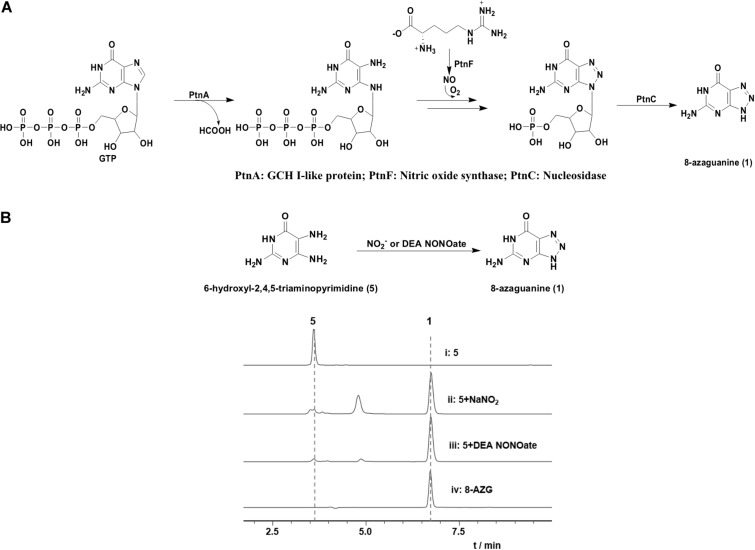
**(A)** Proposed Ptn proteins catalytic pathway. **(B)** HPLC analysis of the reaction mixtures of 5 (0.5 mM) with NaNO_2_ (10 mM) or DEA NONOate (1 mM) incubated for 1 h at room temperature in H_2_O (pH 6.8): (i) 5, (ii) 5 and sodium nitrite, (iii) 5 and DEA NONOate, (iv) 8-AZG standard.

According to the hints of non-enzymatic chemical synthesis, triazole could be produced from diamino with the adding of NO or NO${}_{2}^{-}$ into the reaction system. When we incubated NO generator DEA NONOate or sodium nitrite with 4-hydroxy-2,5,6-triaminopyrimidine (5) at pH 6.8, the production of 8-AZG was detected (Figure 4B). One of the proposed mechanism for triazole formation is NO or NO${}_{2}^{-}$ derivative N_2_O_3_ reacting with an amine at neutral pH to form N-nitrosamine, which can be diazotized and then reacts with the second amine to generate the triazole. Alternatively, a radical intermediate of the diamino derivatives is formed and then reacts with NO to give N-nitrosamine (Kers et al., 2004). The heterologous expression and feeding results suggested that the functions of 8-*azgG* and 8-*azgE* are unable to be substituted with sodium nitrite (Figure 3B and Supplementary Figure 5). We thus proposed two possibilities: one is 8-AzgG and 8-AzgE could be involved in the reaction with NO (or NO${}_{2}^{-}$) to give reactive nitrogen species; the other is that they catalyze the diamino radical intermediate formation, then to afford 1,2,3-triazole. Interestingly, 8-AzgG is homologous to oxygen-independent coproporphyrinogen-III oxidase, which is a family of radical SAM (S-adenosylmethionine) enzyme that has conserved CxxxCxxC three cysteine residues motif to coordinate [4Fe-4S] cluster (Supplementary Figure 21). The [4Fe-4S] cluster binds SAM and cleaves its carbon sulfur bond to yield a highly reactive 5′-deoxyadenosyl (Ado) radical, which then initiates a series of radical reactions (Zhang et al., 2012). Therefore, 8-AzgG could be the candidate to trigger radical intermediate formation. The feeding of 5 into 8-azgABDG heterologous expression broth did not form detectable 8-AzgG, which implies the possibility of substrate selectivity in 8-AZG triazole enzymatic reaction or 5 could not be fed into the *S. albus* strain. Unfortunately, we have not obtained the soluble 8-AzgG protein for the *in vitro* test yet.

Next, 8-AzgD was overexpressed in *E. coli*, and the purified protein was incubated with 2 and 3, but no new product was detected. Based on the sequence homology analysis, 8-azgD belongs to the family of cytokinin riboside 5′-monophosphate phosphoribohydrolase, which catalyzes the hydrolytic removal of ribose 5′-monophosphate (Kurakawa et al., 2007). Therefore, we speculated 8-AzgD catalyzes the last step of 8-AZG biosynthesis. The authentic substrate of 8-AzgD, 5′-monophosphate 8-azaguanosine is rapidly converted into 2 and 3 by the metabolic system of *S. pathocidicus*. Another enzyme 8-AzgE has little homology with other known proteins, and interestingly, the purified protein has a visible pink color, which means the binding of metal ion. The function of 8-AzgE is still a mystery.

## Discussion

**FIGURE 5:**
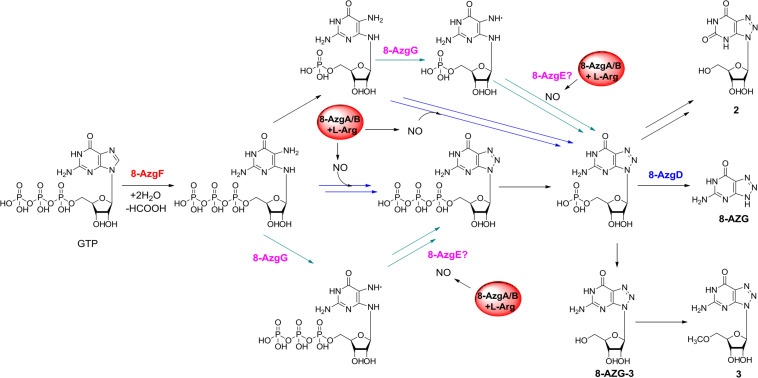
Proposed biosynthetic pathway to 8-azaguanine.

On the basis of the *in vivo* and *in vitro* analysis, we propose a complete 8-AZG biosynthetic pathway as follows: imidazole ring of the starting unit GTP was hydrolytic opened by 8-AzgF to produce 2,5-diamino-6-ribosylamino-4(*3H*)-pyrimidinone 5′-triphosphate. Then, the 2,5-diamino-6-ribosylamino-4(*3H*)-pyrimidinone 5′-triphosphate or a radical intermediate formed by 8-AzgG is converted to 5′-triphosphate 8-azaguanosine by reactive nitrogen species, which is transformed from arginine by the synergistic effect of 8-AzgA and 8-AzgB followed by dephosphorylating conversion to 5′-monophosphate 8-azaguanosine. Alternatively, 2,5-diamino-6-ribosylamino-4(*3H*)-pyrimidinone 5′-triphosphate is dephosphorylated into 2,5-diamino-6-ribosylamino-4(*3H*)-pyrimidinone 5′-monophosphate or converted into 5′-monophosphate radical intermediate, which is activated by reactive nitrogen species to form 5′-monophosphate 8-azaguanosine. The final hydrolytic removal of ribose 5′-monophosphate is catalyzed by 8-AzgD to afford 8-AZG (Figure 5).

The research of Zhao et al. also proposed a biosynthetic route to 8-AZG, which is basically the same as our proposed pathway without the participation of 8-AzgG and 8-AzgE (Figure 4A). In the beginning, the GCH I–like enzyme PtnA catalyzes the hydrolytic cleavage of GTP. The resulting product 2,5-diaminopyrimidine triphosphate could combine with an NO-derived reactive species to afford 8-azaguanosine triphosphate, followed by pyrophosphate release by host enzyme. Alternatively, the compound 2,5-diaminopyrimidine triphosphate could undergo pyrophosphate release and then be followed by triazole formation to afford 5′-monophosphate 8-azaguanosine. The last step could be the 5′-monophosphate 8-azaguanosine hydrolytic removal of ribose 5′-monophosphate to 1 by PtnC, and they carried out *in vitro* test to prove the activity of PtnC as a monophosphate nucleosidase (Zhao et al., 2020). The work of Zhao et al. reported some similar results and also plenty of differences as those presented here. First, they used heterologous expression to locate the biosynthetic gene cluster, for which we adopted both native strain gene knockout and heterologous expression methods. Five additional genes were disrupted, and two undiscussed genes 8-*azgB/G* could be important for 8-AZG biosynthesis. Second, we identified two new metabolite products 2 and 3 from the Δ8-*azgD* mutant strain fermentation broth; however, the compound 1,2,3-triazole nucleoside 8-azaguanosine was not accumulated in our mutant or heterologous expression strains. There are also some new findings in the heterologous gene expression and feeding experiments, such as the proposed 8-*azgA/B* reactive nitrogen species generation or transportation part. Even though the NO and NO${}_{2}^{-}$ involved non-enzymatic production of 8-AZG has been confirmed (Ding et al., 2012), based on the *in vivo* data, a more efficient enzymatic pathway should be existed in the 8-AZG biosynthesis. 8-AzgG and 8-AzgE are likely to participate in the 1,2,3-triazole cascade enzyme catalysis process, but their true functions still need to be explored.

In summary, we have clearly identified the entire biosynthetic gene cluster of 8-AZG by genome scanning, targeted gene deletion, heterologous expression, and feeding experiments, which sets the stage for scrutinizing the chemical logic and enzymatic machinery in 8-AZG biosynthesis. The isolation and structural elucidation of several metabolites and bioinformatics analysis shed light on the functions and timing of some enzymes. We further revealed the NO and reactive nitrogen species–related genes in triazole biosynthesis and proved the non-enzymatic ring formation reaction of 8-AZG 1,2,3-triazole. An enzymatic pathway for triazole construction was proposed according to the *in vivo* experiments. Additional genetic and biochemical studies will provide more insight into the timing and mechanism of the biosynthetic enzymes in producing 8-AZG and promote the engineered biosynthesis of new 1,2,3-triazole-type compounds for drug discovery.

## Data Availability Statement

The DNA sequence of the 8-azg gene cluster has been deposited in GenBank under the accession number MT543149.

## Author Contributions

The manuscript was written through contributions of all authors. All authors have given approval to the final version of the manuscript. WH designed the research plan. FH, YW, and QG performed the experiments. WH, MX, and FH analyzed the data. WH, MX, and FH wrote the manuscript.

## Conflict of Interest

The authors declare that the research was conducted in the absence of any commercial or financial relationships that could be construed as a potential conflict of interest.

## Footnotes

1 http://nocardia.nih.go.jp/fp4/

2 http://blast.ncbi.nlm.nih.gov/Blast.cgi

## References

[B1] Anzai K. Nagatsu J. Suzuki S. (1961). Pathocidin, a new antifungal antibiotic, I. isolation, physical and chemical properties, and biological activities. *J. Antibiot.* 14 340–342. 14036815

[B2] Choudhary A. Zachek B. Lera R. F. Zasadil L. M. Lasek A. Denu R. A. (2016). Identification of selective lead compounds for treatment of High-Ploidy breast cancer. *Mol. Cancer Ther.* 15 48–59. 10.1158/1535-7163.MCT-15-0527 26586723 PMC4707107

[B3] Ding Z. German P. Bai S. Feng Z. Gao M. Si W. (2012). Agents That stabilize mutated von Hippel-Lindau (VHL) protein: results of a high-throughput screen to identify compounds that modulate VHL proteostasis. *J. Biomol. Screen* 17 572–580. 10.1177/1087057112436557 22357874 PMC3895461

[B4] Du Y.-L. He H.-Y. Higgins M. A. Ryan K. S. (2017). A heme-dependent enzyme forms the nitrogen-nitrogen bond in piperazate. *Nat. Chem. Biol.* 13 836–838. 10.1038/nchembio.2411 28628093

[B5] Goddard A. D. Moir J. W. B. Richardson D. J. Ferguson S. (2008). Interdependence of two NarK domains in a fused nitrate/nitrite transporter. *J. Mol. Microbiol.* 70 667–681. 10.1111/j.1365-2958.2008.06436.x 18823285

[B6] Guo Y.-Y. Li Z.-H. Xia T.-Y. Du Y.-L. Mao X.-M. Li Y.-Q. (2019). Molecular mechanism of azoxy bond formation for azoxymycins biosynthesis. *Nat. Commun.* 10:4420. 10.1038/s41467-019-12250-1 31594923 PMC6783550

[B7] He H.-Y. Henderson A. C. Du Y.-L. Ryan K. S. (2019). Two-enzyme pathway links L-arginine to Nitric Oxide in N-Nitroso biosynthesis. *J. Am. Chem. Soc.* 141 4026–4033. 10.1021/jacs.8b13049 30763082

[B8] Hirasawa K. Isono K. (1978). Formation of 8-azaguanine from guanine by *Streptomyces albus*. *J. Antibiot.* 31 628–629. 10.7164/antibiotics.31.628 681246

[B9] Kers J. A. Wach M. J. Krasnoff S. B. Widom J. Cameron K. D. Bukhalid R. A. (2004). Nitration of a peptide phytotoxin by bacterial nitric oxide synthase. *Nature* 429 79–82. 10.1038/nature02504 15129284

[B10] Kim N. Choi J. W. Song A. Y. Choi W. S. Park H. R. Park S. (2019). Direct potentiation of NK cell cytotoxicity by 8-azaguanine with potential antineoplastic activity. *Int. Immunopharmacol.* 67 152–159. 10.1016/j.intimp.2018.12.020 30551032

[B11] Kurakawa T. Ueda N. Maekawa M. Kobayashi K. Kojima M. Nagato Y. (2007). Direct control of shoot meristem activity by a cytokinin-activating enzyme. *Nature* 445 652–655. 10.1038/nature05504 17287810

[B12] Matsuda K. Tomita T. Shin-ya K. Wakimoto T. Kuzuyama T. Nishiyama M. (2018). Discovery of unprecedented hydrazine-forming machinery in bacteria. *J. Am. Chem. Soc.* 140 9083–9086. 10.1021/jacs.8b05354 30001119

[B13] Möller M. N. Rios N. Trujillo M. Radi R. Denicola A. Alvarez B. (2019). Detection and quantification of nitric oxide-derived oxidants in biological systems. *J. Biol. Chem.* 294 14776–14802. 10.1074/jbc.REV119.006136 31409645 PMC6779446

[B14] Ng T. L. Rohac R. Mitchell A. J. Boal A. K. Balskus E. P. (2019). An N-nitrosating metalloenzyme constructs the pharmacophore of streptozotocin. *Nature* 566 94–99. 10.1038/s41586-019-0894-z 30728519 PMC6369591

[B15] Rebelo J. Auerbach G. Bader G. Bracher A. Nar H. Hösl C. (2003). Biosynthesis of pteridines. reaction mechanism of GTP cyclohydrolase I. *J. Mol. Biol.* 326 503–516. 10.1016/S0022-2836(02)01303-7 12559918

[B16] Sao Emani C. Williams M. J. Wiid I. J. Baker B. Carolis C. (2018). Compounds with potential activity against *Mycobacterium tuberculosis*. *Antimicrob. Agents* 62:e02236-17. 10.1128/aac.02236-17 29437626 PMC5913991

[B17] Sugai Y. Katsuyama Y. Ohnishi Y. (2016). A nitrous acid biosynthetic pathway for diazo group formation in bacteria. *Nat. Chem. Biol.* 12 73–75. 10.1038/nchembio.1991 26689788

[B18] Twigg F. F. Cai W. Huang W. Liu J. Sato M. Perez T. J. (2019). Identifying the biosynthetic gene cluster for triacsins with an N-Hydroxytriazene moiety. *Chembiochem* 20 1145–1149. 10.1002/cbic.201800762 30589194 PMC6590916

[B19] Waldman A. J. Pechersky Y. Wang P. Wang J. X. Balskus E. P. (2015). The cremeomycin biosynthetic gene cluster encodes a pathway for diazo formation. *Chembiochem* 16 2172–2175. 10.1002/cbic.201500407 26278892 PMC4996270

[B20] Wang K.-K. A. Ng T. L. Wang P. Huang Z. Balskus E. P. van der Donk W. A. (2018). Glutamic acid is a carrier for hydrazine during the biosyntheses of fosfazinomycin and kinamycin. *Nat. Commun.* 9:3687. 10.1038/s41467-018-06083-7 30206228 PMC6133997

[B21] Xia M. Bi K. Huang R. Cho M.-H. Sakamuru S. Miller S. C. (2009). Identification of small molecule compounds that inhibit the HIF-1 signaling pathway. *Mol. Cancer* 8:117. 10.1186/1476-4598-8-117 20003191 PMC2797767

[B22] Zhang Q. van der Donk W. A. Liu W. (2012). Radical-mediated enzymatic methylation: a tale of two SAMS. *Acc. Chem. Res.* 45 555–564. 10.1021/ar200202c 22097883 PMC3328197

[B23] Zhao G. Guo Y.-Y. Yao S. Shi X. Lv L. Du Y.-L. (2020). Nitric oxide as a source for bacterial triazole biosynthesis. *Nat. Commun.* 11:1614. 10.1038/s41467-020-15420-8 32235841 PMC7109123

