# Supplementary material for: Identification of 8-Azaguanine Biosynthesis–Related Genes Provides Insight Into the Enzymatic and Non-enzymatic Biosynthetic Pathway for 1,2,3-Triazole
Source: Front Bioeng Biotechnol. 2020 Nov 5;8:603514. doi: 10.3389/fbioe.2020.603514 (PMC7674941; doi:10.3389/fbioe.2020.603514)
Supplement: Supplementary file 1 [file Table_1.DOCX]

Supplementary Material

**Table S1.** Comparison of the biosynthetic gene clusters of 8-azaguanine in *S. albus pathocidicus* CGMCC4.1633 and *S. albus* subsp*. pathocidicus* ATCC 14510.

| Gene | Size(aa) | (Putative) function |
| --- | --- | --- |
| 8*-azgA/ptnF* | 390 | Nitric oxide synthase oxygenase |
| 8*-azgB/ptnE* | 408 | MFS transporter |
| 8-*azgC/ptnD* | 236 | ADP-ribose pyrophosphatase |
| 8*-azgD/ptnC* | 167 | Nucleotide monophosphate nucleosidase |
| 8-*azgE/ptnB* | 106/111 | Hypothetical protein |
| 8*-azgF/ptnA* | 206 | GTP cyclohydrolase I |
| 8*-azgG* | 462 | Oxygen-independent coproporphyrinogen-III oxidase-like protein |
| 8*-azgH* | 378 | DUF1152 domain-containing protein |
| 8*-azgI* | 682 | Hypothetical protein |
| 8*-azgJ* | 103 | Hypothetical protein |
| 8*-azgK* | 277 | Glycosyltransferase |

NCBI accession number: MT543149; MN707952 or NZ_LIQY01000068.1 (7148. .11814)

**Table S2.** Primers used in this study.

| pETduet8-AzgAF | TAAGGATCCGGTGAGTCCGGAGGAGGAGCG | Used for 8-AzgA expression |
| --- | --- | --- |
| pETduet8-AzgAR | TAACTCGAGCGCCTGGGCGCGACTTTTGA |  |
| 8-azgleftF | TAAAAGCTT GGTGAGCCCACAAGTGGTAG | Used for ∆1633-*azg* biosynthetic cluster mutant construction |
| 8-azgleftR | TAATCTAGAAGGGAGACCGCGATCAGACC |  |
| 8-azgrightF | TAATCTAGAACCGATCCGCCGGAGGTGGT |  |
| 8-azgrightR | TAAGAATTCTGATGTCCACCTTCATGGAG |  |
| 8-azgAleftF | TAAAAGCTTCGCTCCACGCAGTACACCAG | Used for ∆8-*azgA* mutant construction |
| 8-azgAleftR | TAATCTAGACCTGGGCGCGACTTTTGATG |  |
| 8-azgArightF | TAATCTAGAGCGCTCCTCCTCCGGACTCAC |  |
| 8-azgArightR | TAAGAATTCACTACCGGAAGCTGTTCGGT |  |
| 8-azgBleftF | TAAAAGCTTCACGTCGAGGAGTTCGGTAC | Used for ∆8-*azgB* mutant construction |
| 8-azgBleftR | TAATCTAGACACAGGCGAACCCCGTGAGT |  |
| 8-azgBrightF | TAATCTAGATGGTGTGAGCGGTGTGGTCAT |  |
| 8-azgBrightR | TAAGAATTCTGCTTACGTATGCGTGTACCT |  |
| 8-azgDleftF | TAAAAGCTTTGAGTCGGCCGGTGCCTTCG | Used for ∆8-*azgD* mutant construction |
| 8-azgDleftR | TAATCTAGAGATCTCGGGTAGCCGGCGTG |  |
| 8-azgDrightF | TAATCTAGACATGGGGCCTCAGTTCTTCTT |  |
| 8-azgDrightR | TAAGAATTCAAGTTACGAGTCGAGCGGGT |  |
| 8-azgEleftF | TAAAAGCTTTTCTGTCACCGTGGCTGTTTG | Used for ∆8-*azgE* mutant construction |
| 8-azgEleftR | TAATCTAGATGAGGCCCCATGGAGAAATG |  |
| 8-azgErightF | TAATCTAGACACGGACGCCGACGGTGCTC |  |
| 8-azgErightR | TAAGAATTCTACCGCAGACACCCCGTATG |  |
| 8-azgFleftF | TAAAAGCTTGTCGACAAACGCCGGGTAGC | Used for ∆8-*azgF* mutant construction |
| 8-azgFleftR | TAATCTAGAAGATAGGCGAGCCGAATGGAC |  |
| 8-azgFrightF | TAATCTAGACATGGTGTCATCCTCCAGGTC |  |
| 8-azgFrightR | TAAGAATTCTGGCAGGTTGATTCTCTGCGT |  |
| 8-azgGleftF | TAAGAATTCCGAACAGGTGGAACACCGAG | Used for ∆8-*azgG* mutant construction |
| 8-azgGleftR | TAATCTAGAGAAGCCATGGCGAGCTACCAG |  |
| 8-azgGrightF | TAATCTAGACATGGACAACACCTTCCCTCT |  |
| 8-azgGrightR | TAAAAGCTTGATGGCCTGGACGAGTTCGT |  |
| 8-azgleftMF | GGTGACCTTCGGGACCGT | Used to verify the ∆1633-*azg* mutant |
| 8-azgleftMR | ACATCTGATCGGTGAATG |  |
| 8-azgAleftMF | GCAGCAGCTTCGGCACCT | Used to verify the ∆8-*azgA* mutant |
| 8-azgAleftMR | CCTGACCTTCCTGCTCTC |  |
| 8-azgBleftMF | CGTGATATCGACGCTGAC | Used to verify the ∆8-*azgB* mutant |
| 8-azgBleftMR | CAGAGGACGCTACGTAAG |  |
| 8-azgDleftMF | TGTGGTGCGGCGGGAAGC | Used to verify the ∆8-*azgD* mutant |
| 8-azgDleftMR | CAGTGCGCACTGCTGCAT |  |
| 8-azgEleftMF | GAGAGTTCGCCCGAGGAC | Used to verify the ∆8-*azgE* mutant |
| 8-azgEleftMR | TGACAAGAGCGAGGTGAT |  |
| 8-azgFleftMF | ACGGTCCCTCCCACCTCG | Used to verify the ∆8-*azgF* mutant |
| 8-azgFleftMR | AACGGCGCCGGATGTATG |  |
| 8-azgGleftMF | GTCGTTGACCAGGATCAG | Used to verify the ∆8-*azgG* mutant |
| 8-azgGleftMR | GCAGTATTCGGCGGCTTG |  |
| 8-azgABCDEFGHf1 | agcttgggctgcaggtcgactctagaCTGCTGCTCGCCGGTCTGAT | Used for 8-*azgABCDEFGH* mutant construction |
| 8-azgABCDEFGHr1 | agataggcgagccGAATGGACCGAGGAGCACCG |  |
| 8-azgABCDEFGHf2 | tccattcGGCTCGCCTATCTCCCCAGG |  |
| 8-azgABCDEFGHr2 | tcgcgcgcggccgcggatcctctagaCAGACCGGGATCCCCACAAG |  |
| 8-azgABCDEFGHf1 | agcttgggctgcaggtcgactctagaCTGCTGCTCGCCGGTCTG AT | Used for 8-*azgACDEFGH* mutant construction |
| 8-azgACDEFGHr1 | tcatcctgacACGTACTTCCTGGCCGGAGC |  |
| 8-azgACDEFGHf2 | ggaagtacgtGTCAGGATGACGGCGCTCAG |  |
| 8-azgABCDEFGHr2 | tcgcgcgcggccgcggatcctctagaCAGACCGGGATCCCCACAAG |  |
| 8-azgBCDEFGHf | gggctgcaggtcgactctagaGTCACGAACTCCTCCGCCAG | Used for 8-*azgBCDEFGH* mutant construction |
| 8-azgBCDEFGHr | CgcggccgcggatcctctagaCAGACCGGGATCCCCACAAG |  |
| 8-azgCDEFGHf | gggctgcaggtcgactctagaGTCAGGATGACGGCGCTCAG | Used for 8-*azgCDEFGH* mutant construction |
| 8-azgCDEFGHr | cgcggccgcggatcctctagaCAGACCGGGATCCCCACAAG |  |
| 8-azgABCDEFGHf1 | agcttgggctgcaggtcgactctagaCTGCTGCTCGCCGGTCTGAT | Used for 8-*azgABCDEF* mutant construction |
| 8-azgABCDEFr | tcgcgcgcggccgcggatcctctagaGCACCACACGAACACCTGGC |  |
| 8-azgABCDEFGHf1 | agcttgggctgcaggtcgactctagaCTGCTGCTCGCCGGTCTGAT | Used for 8-*azgABF* mutant construction |
| 8-azgABFr | tcgcgcgcggccgcggatcctctagaGCACCACACGAACACCTGGC |  |
| 8-azgABCDEFGHf1 | agcttgggctgcaggtcgactctagaCTGCTGCTCGCCGGTCTGAT | Used for 8-*azgABFG* mutant construction |
| 8-azgABFGr1 | caccgtcCACTGGGGAGCTTCCTCGTG |  |
| 8-azgABFGf2 | aagctccccagtgGACGGTGCTCCTCGGTCC |  |
| 8-azgABCDEFGHr2 | tcgcgcgcggccgcggatcctctagaCAGACCGGGATCCCCACAAG |  |
| 8-azgABCDEFGHf1 | agcttgggctgcaggtcgactctagaCTGCTGCTCGCCGGTCTGAT | Used for 8-*azgABDG* mutant construction |
| 8-azgABCDGr1 | tgagCACTGGGGAGCTTCCTCGTG |  |
| 8-azgABCDGf2 | aggaagctccccagtgCTCAGCGGGCACTCCTTGT |  |
| 8-azgABCDGr2 | aaatGAGAGCACCCCGACAACTCG |  |
| 8-azgABCDGf3 | ttgtcggggtgctctcATTTCGTGGCCTCTCCGTG |  |
| 8-azgABCDEFGHr2 | tcgcgcgcggccgcggatcctctagaCAGACCGGGATCCCCACAAG |  |

**Table S3.** Strains and plasmids used and generated in this study.

| **Strain/ plasmid** | **Purpose** | **Sources** |
| --- | --- | --- |
| **Strains** | | |
| *E. coli* | | |
| BL21(DE3)- pGr07 | For protein expression | Invitrogen |
| DH5α | For plasmids construction | Invitrogen |
| S17-1 | Donor strain for conjugation | Invitrogen |
| *Streptomyces* | | |
| *Streptomyces albus subsp. pathocidicus* CGMCC4.1633 | 8-Azg wild type producing strain | CGMCC |
| ∆8-*azgABCDEFGHIJK* | 8-*azgABCDEFGHIJK* inactivation mutant of *Streptomyces albus subsp. pathocidicus* CGMCC4.1633 | This study |
| ∆8-*azgA* | 8-*azgA* inactivation mutant of *Streptomyces albus subsp. pathocidicus* CGMCC4.1633 | This study |
| ∆8-*azgB* | 8-*azgB* inactivation mutant of *Streptomyces albus subsp. pathocidicus* CGMCC4.1633 | This study |
| ∆8-*azgD* | 8-*azgD* inactivation mutant of *Streptomyces albus subsp. pathocidicus* CGMCC4.1633 | This study |
| ∆8-*azgE* | 8-*azgE* inactivation mutant of *Streptomyces albus subsp. pathocidicus* CGMCC4.1633 | This study |
| ∆8-*azgF* | 8-*azgF* inactivation mutant of *Streptomyces albus subsp. pathocidicus* CGMCC4.1633 | This study |
| ∆8-*azgG* | 8-*azgG* inactivation mutant of *Streptomyces albus subsp. pathocidicus* CGMCC4.1633 | This study |
| 8-*azgABCDEFGH* | 8-AZG heterologous expression in *S. albus* J1074. | This study |
| 8-*azgBCDEFGH* | 8-AZG heterologous expression in *S. albus* J1074. | This study |
| 8*-azgCDEFGH* | 8-AZG heterologous expression in *S. albus* J1074. | This study |
| 8-*azgACDEFGH* | 8-AZG heterologous expression in *S. albus* J1074. | This study |
| 8-*azgABCDEF* | 8-AZG heterologous expression in *S. albus* J1074. | This study |
| 8-*azgABFG* | 8-AZG heterologous expression in *S. albus* J1074. | This study |
| 8-*azgABDG* | 8-AZG heterologous expression in *S. albus* J1074. | This study |
| 8-*azgABF* | 8-AZG heterologous expression in *S. albus* J1074. | This study |
| **Plasmids** | | |
| pET-DUET | Amp^r^, vector for protein expression | Invitrogen |
| pKC1139 | Apr^r^, vector for conjuction | Ref ^2^ |
| pSET152 | Apr^r^, vector for conjuction | Invitrogen |
| pETduet8-AzgA | Amp^r^ , for over-expression of 8-AzgA | This study |
| pKC1139*azgABCDEFGHIJK* | Apr^r^, gene inactivation plasmid used for 8-*azgABCDEFGHIJK* mutant construction | This study |
| pKC1139*azgA* | Apr^r^, gene inactivation plasmid used for 8-*azgA* mutant construction | This study |
| pKC1139*azgB* | Apr^r^, gene inactivation plasmid used for 8-*azgB* mutant construction | This study |
| pKC1139*azgD* | Apr^r^, gene inactivation plasmid used for 8-*azgD* mutant construction | This study |
| pKC1139*azgE* | Apr^r^, gene inactivation plasmid used for 8-*azgE* mutant construction | This study |
| pKC1139*azgF* | Apr^r^, gene inactivation plasmid used for 8-*azgF* mutant construction | This study |
| pKC1139*azgG* | Apr^r^, gene inactivation plasmid used for 8-*azgG* mutant construction | This study |
| pSET*azgABCDEFGH* | Apr^r^, gene integration plasmid used for 8-*azgABCDEFGH* mutant construction | This study |
| pSET*azgABFG* | Apr^r^, gene integration plasmid used for 8-*azgABFG* mutant construction | This study |
| pSET*azgBCDEFGH* | Apr^r^, gene integration plasmid used for 8-*azgBCDEFGH* mutant construction | This study |
| pSET*azgACDEFGH* | Apr^r^, gene integration plasmid used for 8-*azgACDEFGH* mutant construction | This study |
| pSET*azgCDFGH* | Apr^r^, gene integration plasmid used for 8-*azgCDEFGH* mutant construction | This study |
| pSET*azgABCDEF* | Apr^r^, gene integration plasmid used for 8-*azgABCDEF* mutant construction | This study |
| pSET*azgABDG* | Apr^r^, gene integration plasmid used for 8-*azgABCDG* mutant construction | This study |
| pSET*azgABF* | Apr^r^, gene integration plasmid used for 8-*azgABF* mutant construction | This study |

**Supplementary Figure 1.** Gene inactivation in *Streptomyces albus subsp. pathocidicus* CGMCC4.1633.


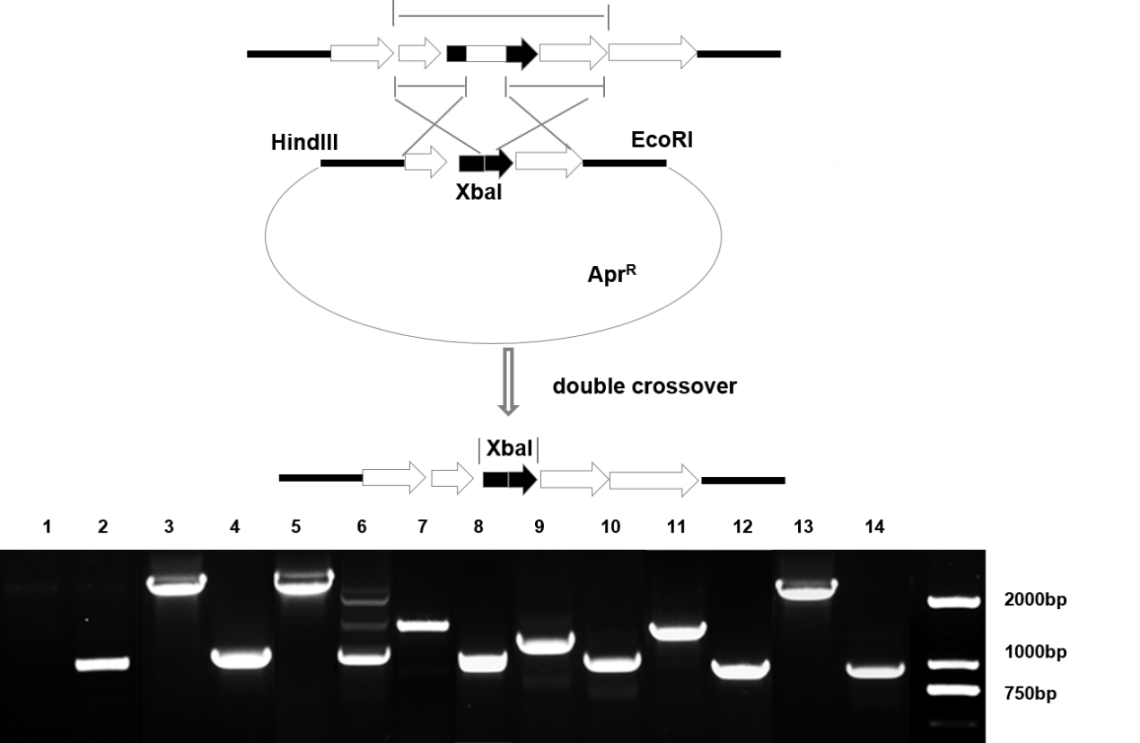


All genes were deleted in-frame by homologous recombination as illustrated above. The genomic DNA of the wild-type and mutant strains were extracted and used as templates for subsequent PCR verification of the mutant. PCR was carried out using two external primers from adjacent genes (Table S2).The consistent with the expected sizes for the mutants. Lane 1: WT(control); lane 2: Δ1633-*azg* 1kb, lane 3: WT 2.1kb; lane 4: Δ8-*azgA* 1kb; lane 5: WT 2.2kb, lane 6: Δ8-*azgB* 1kb; lane 7: WT 1.5kb, lane 8: Δ8-*azgD* 1kb; lane 9: WT 1.3kb, lane 10: Δ8-*azgE* 1kb; lane 11: WT 1.5kb, lane 12: Δ8-*azgF* 1kb; lane 13: WT 2.4kb, lane 14: Δ8-*azgG* 1kb.

**Supplementary Figure 2.** *In vitro* characterization of 8-AzgA.


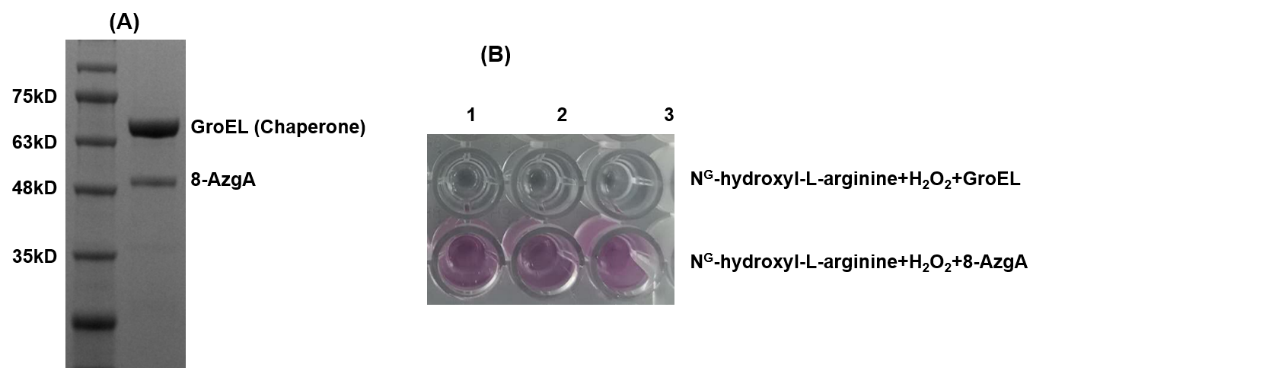


(A) SDS-PAGE of isolated 8-AzgA. Criterion MOPS gel (12% precast, Biorad) was used. (B) Biochemical assay of 8-AzgA. Nitrite production was quantified by using Griess Reagents as described in Methods.

**Supplementary Figure 3.** LC-HRMS analysis of **1** heterologous production in *S. albus* J1074.


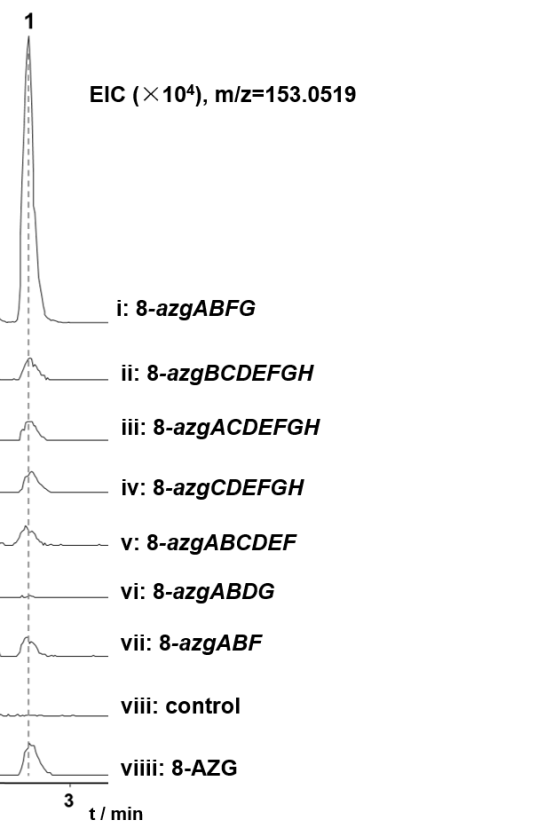


**Supplementary Figure 4.** HPLC analysis with UV detection (245nm) of *Streptomyces albus subsp. pathocidicus* CGMCC4.1633 wild type strain and the mutants.


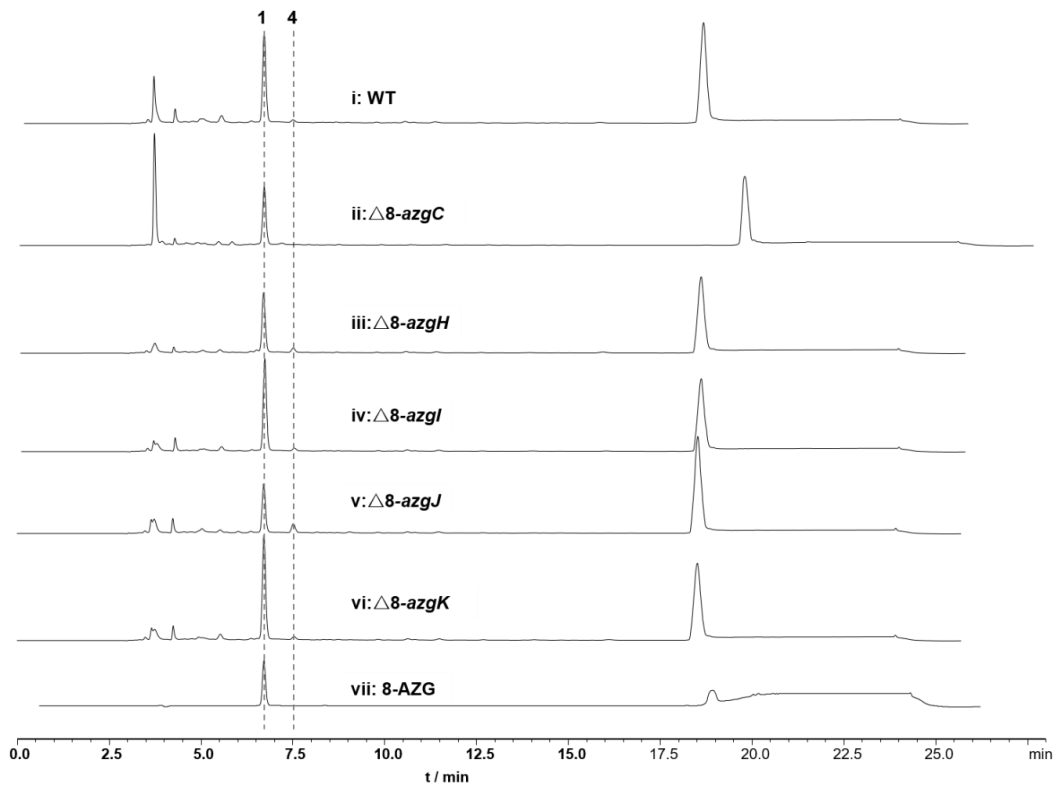


**Supplementary Figure 5.** HPLC analysis of **1** from NaNO_2_ (10mM) feeding experiments in *S. albus* J1074 heterologous expression strains.


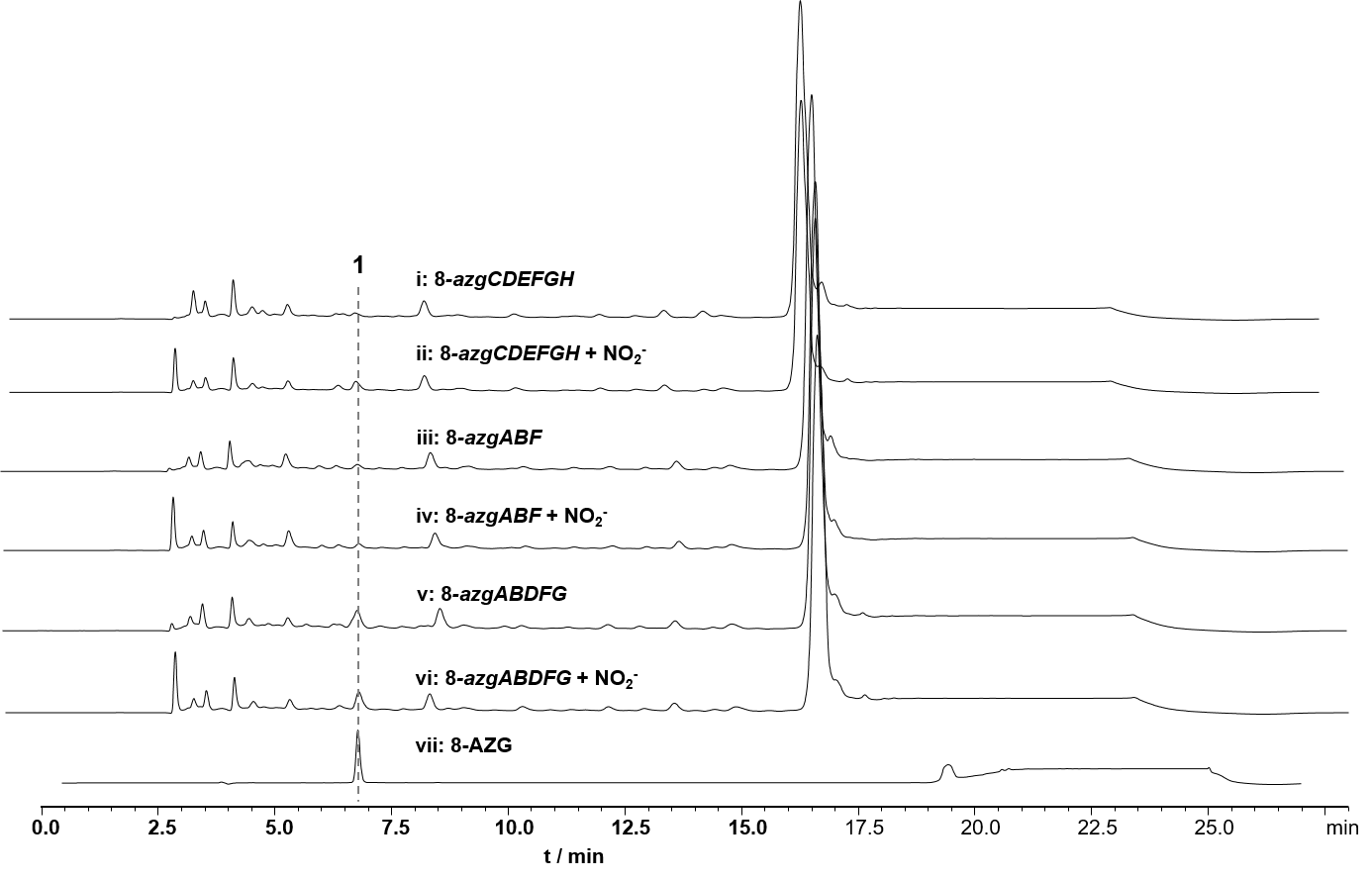


**Supplementary Figure 6.** (A) HPLC analysis of **4** from mutant strain Δ8-*azgD*. i): adenosine standard; ii): mutant strain Δ8-*azgD*. (B) HRMS analysis of **4**.


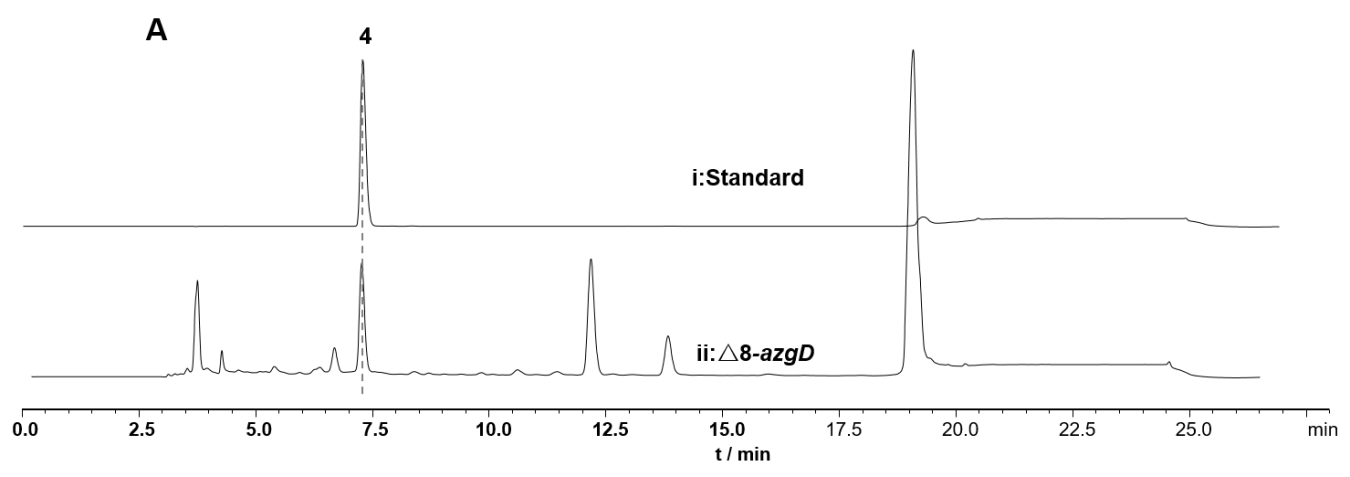


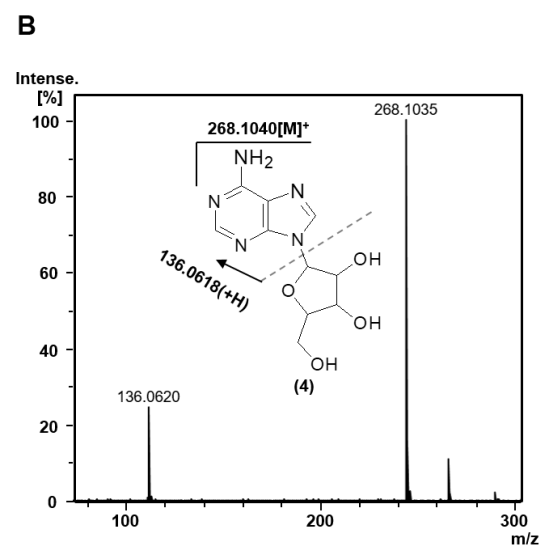


| Formula (M) | Ion Formula | m/z | Calc m/z | Diff (ppm) |
| --- | --- | --- | --- | --- |
| C_10_H_13_N_5_O_4_ | C_10_H_14_N_5_O_4_ | 268.1035 | 268.1040 | 1.87 |

**Table S4.** NMR Data for **2**-**3** (D_2_O, TMS, δ, ppm).

**
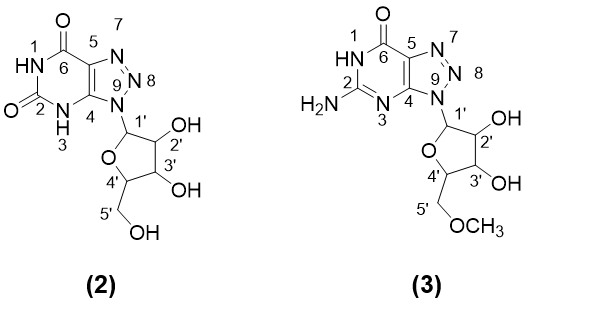
**

| Position | **2** | | **3** | |
| --- | --- | --- | --- | --- |
|  | *δ*_C_*^a^* | *δ*_H_*^b^* (*J* in Hz) | *δ*_C_*^a^* | *δ*_H_*^b^* (*J* in Hz) |
| 2 | 160.4,C |  | 156.2, C |  |
| 4 | 153.4, C |  | 151.9, C |  |
| 5 | 124.1, C |  | 124.4, C |  |
| 6 | 160.2, C |  | 158.4, C |  |
| 1' | 88.7, CH | 6.11 (d, *J* = 4.7 Hz, 1H) | 88.4, CH | 6.17 (d, *J* = 3.6 Hz, 1H) |
| 2' | 73.4, CH | 4.93 (t, *J* = 4.7 Hz, 1H ) | 73.4, CH | 4.98 (dd, *J* = 5.3 Hz, *J* = 3.6 Hz, 1H) |
| 3' | 70.7, CH | 4.56 (t, *J* = 4.7 Hz, 1H) | 70.7, CH | 4.62 (t, *J* = 5.3 Hz, 1H) |
| 4' | 85.5, CH | 4.26 (q, *J* = 4.2 Hz, 1H) | 83.1, CH | 4.33 (td, *J* = 6.2 Hz, *J* = 3.1 Hz, 1H) |
| 5' | 61.6, CH2 | 3.86 (dd, *J* = 12.7 Hz, *J* = 2.9 Hz, 1H ) | 72.1, CH_2_ | 3.66 (dd, *J* = 11.4 Hz, *J* = 6.7 Hz, 1H) |
|  |  | 3.74 (dd, *J* = 12.7 Hz, *J* = 4.2 Hz, 1H ) |  | 3.74 (dd, *J* = 11.4 Hz, *J* = 3.1 Hz, 1H) |
| -OCH_3_ |  |  | 58.7, CH_3_ | 3.34 (1H, s) |
| *^a^*Data collected at 150 MHz; *^b^*Data collected at 600 MHz. | | | | |

**Supplementary Figure 7.** HRMS spectrum and UV of **2**.

**
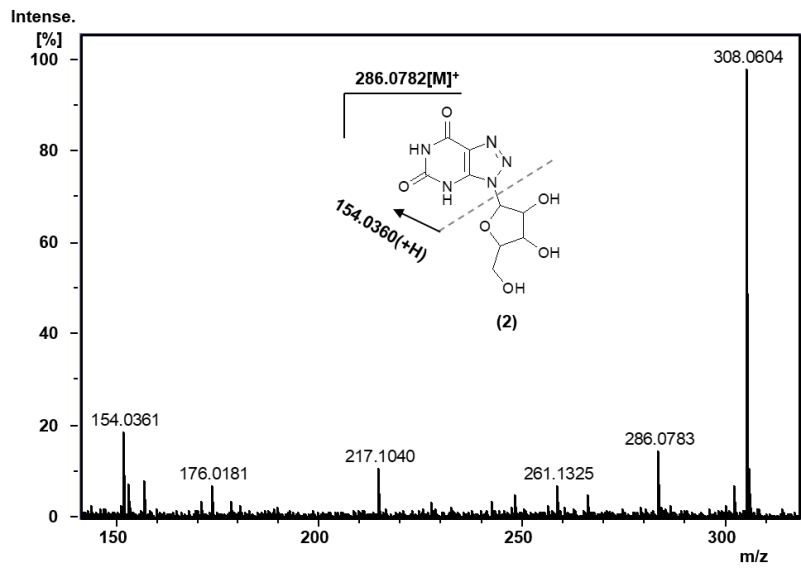
**

| Formula (M) | Ion Formula | m/z | Calc m/z | Diff (ppm) |
| --- | --- | --- | --- | --- |
| C_9_H_11_N_5_O_6_ | C_9_H_12_N_5_O_6_ | 286.0783 | 286.0782 | 0.35 |
|  |  |  |  |  |

**
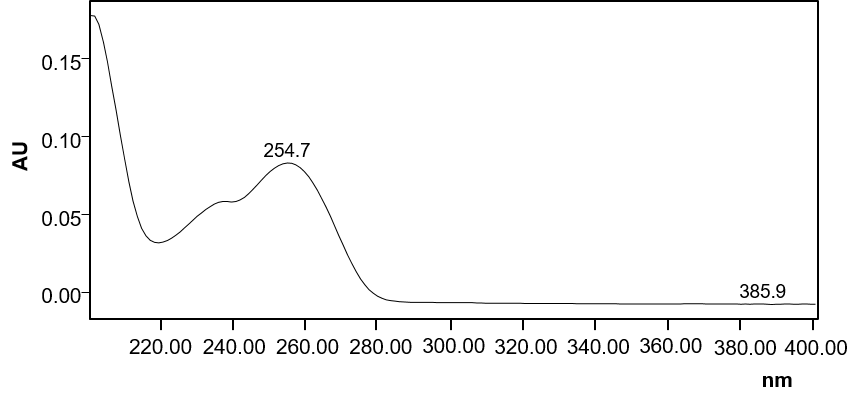
**

**Supplementary Figure 8.** ^1^H NMR (600 MHz) spectrum of **2** in D_2_O.

**
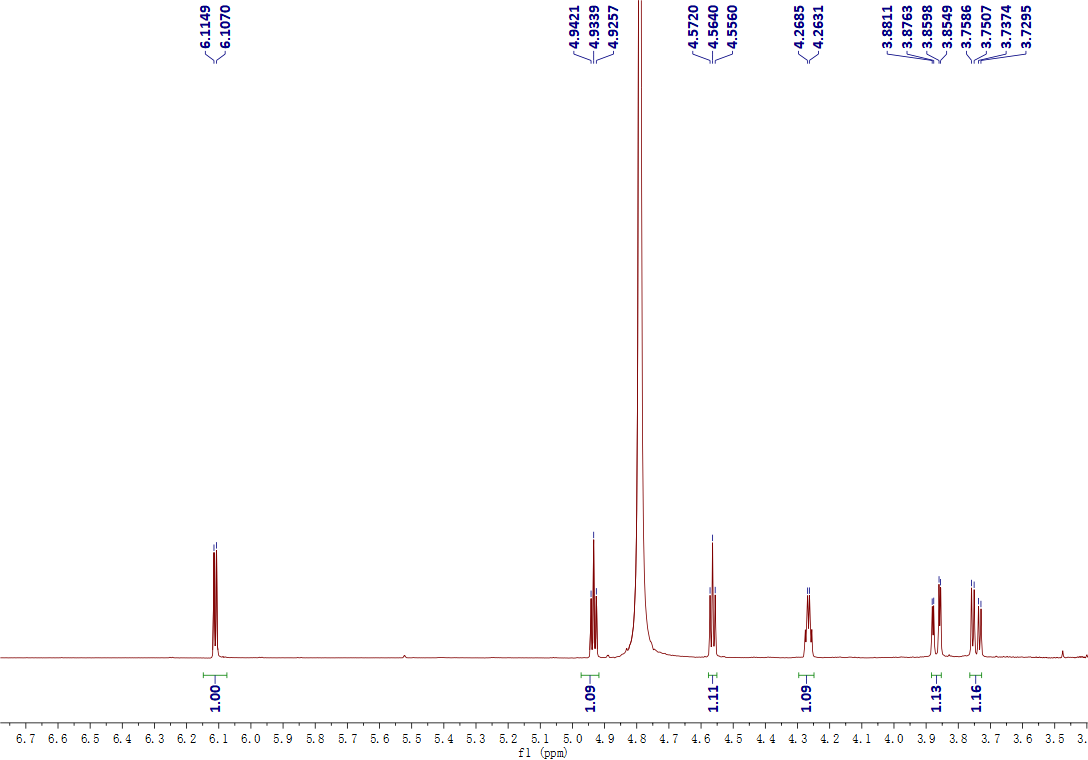
**

**2**

**Supplementary Figure 9.** ^13^C NMR (150 MHz, D_2_O) and DEPT spectra of compound of **2**.

_
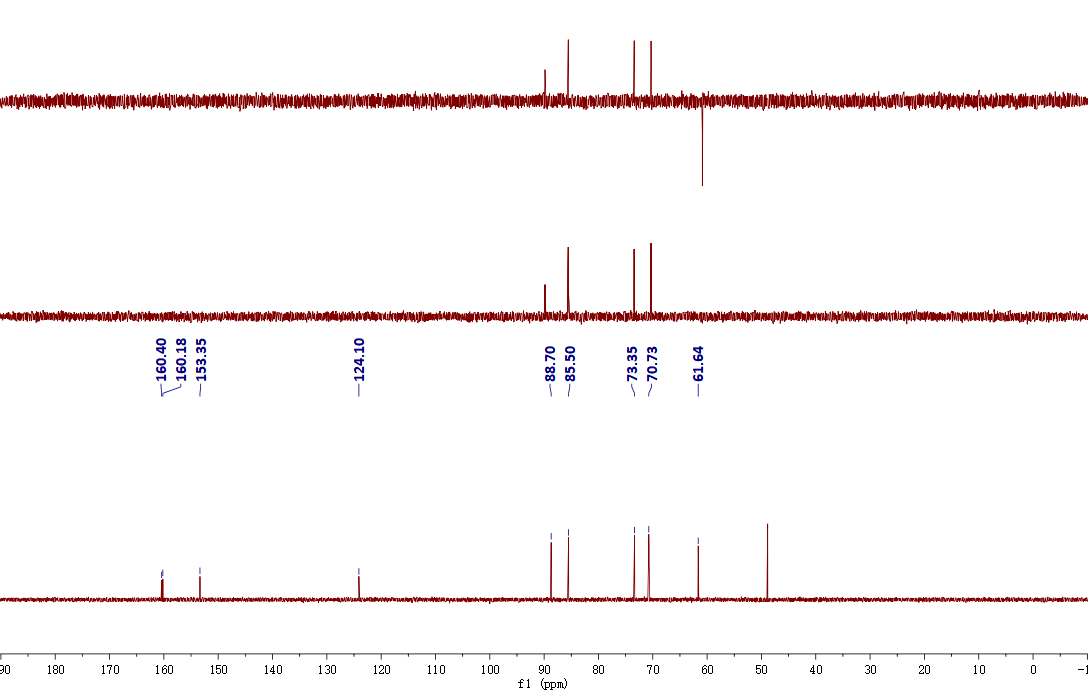
_

×

CH_3_OH

×

**2**

** Supplementary Figure 10.** ^1^H-^1^H COSY spectrum of **2** in D_2_O.


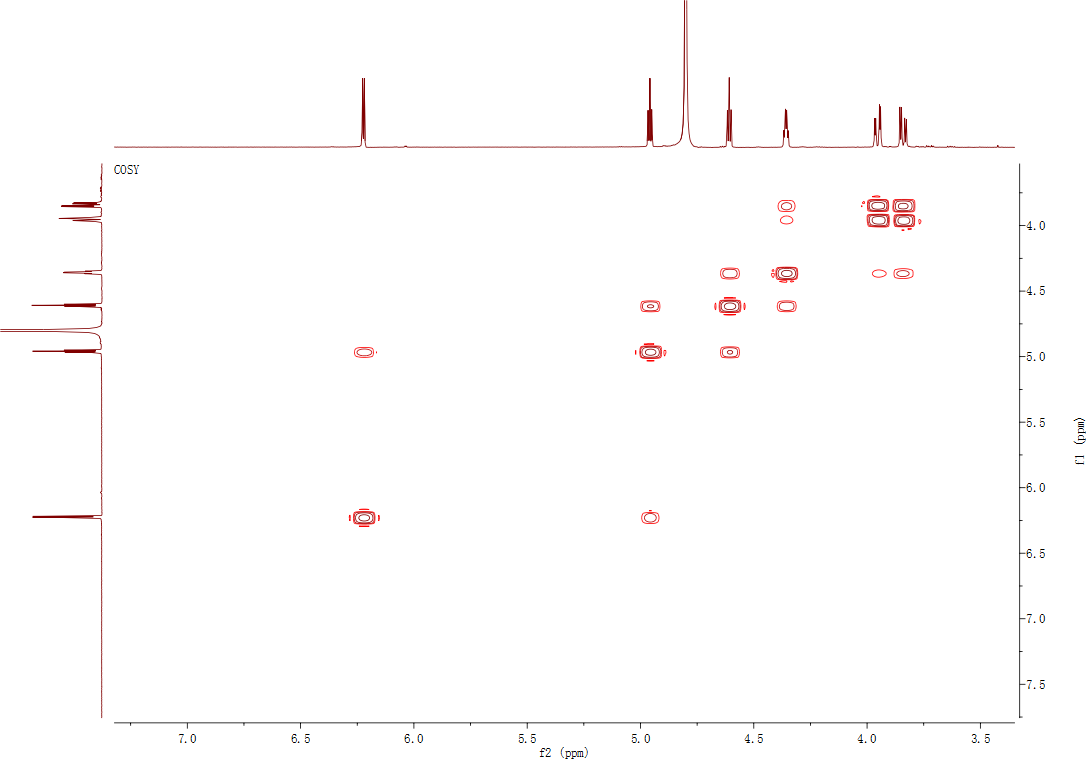


** Supplementary Figure 11.** HSQC spectrum of **2** in D_2_O.


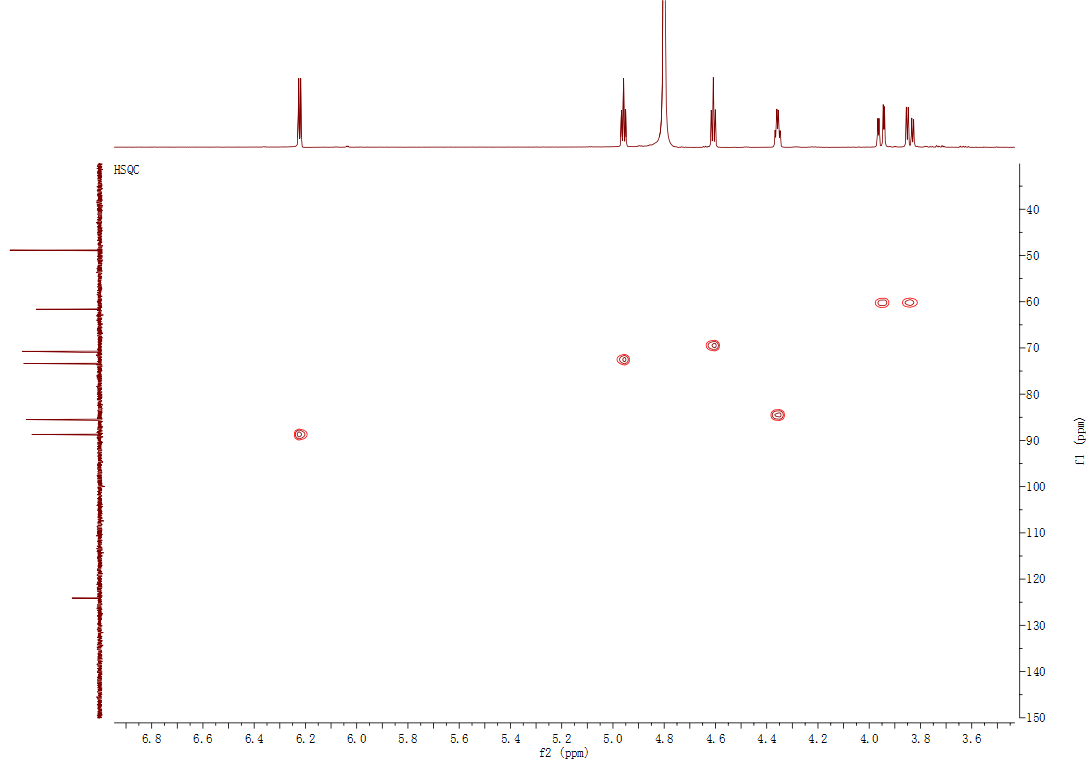


**Supplementary Figure 12.** HMBC spectrum of **2** in D_2_O.


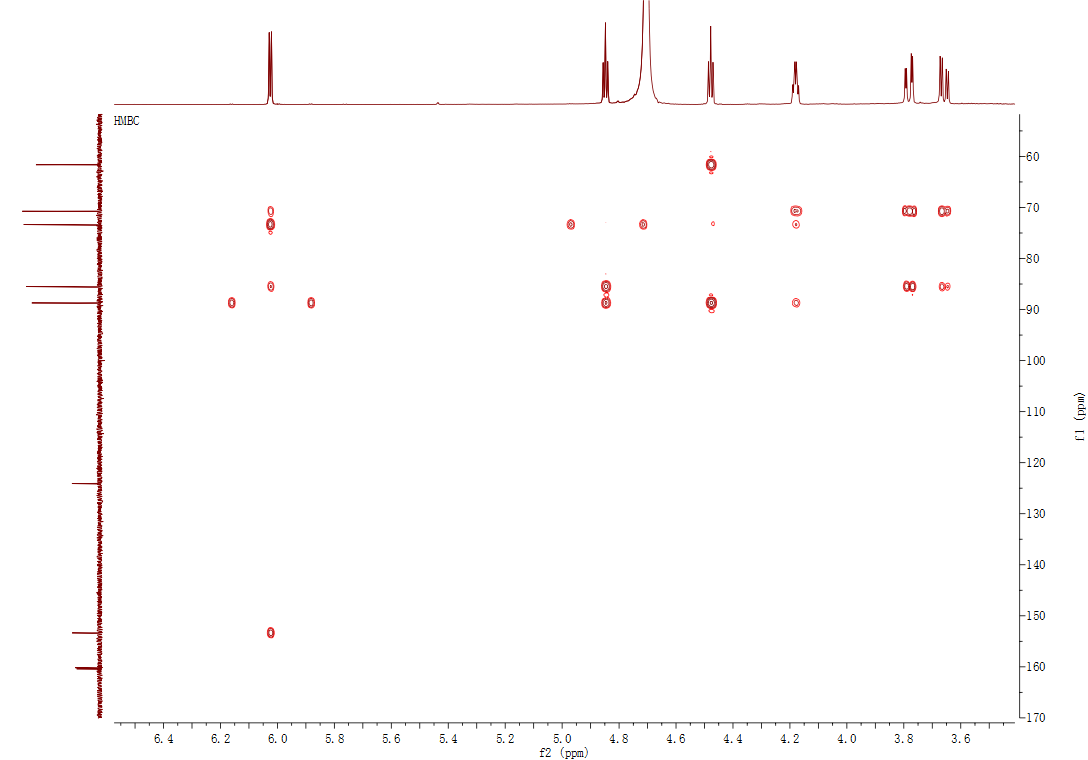

**2**

**Supplementary Figure 13.** HRMS spectrum and UV of **3**.


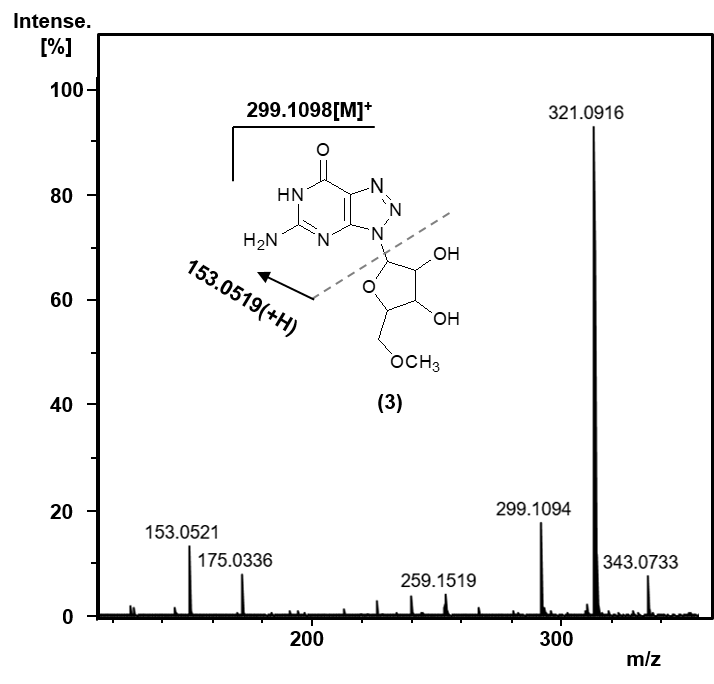


| Formula (M) | Ion Formula | m/z | Calc m/z | Diff (ppm) |
| --- | --- | --- | --- | --- |
| C_10_H_14_N_6_O_6_ | C_10_H_15_N_6_O_6_ | 299.1094 | 299.1098 | 1.33 |


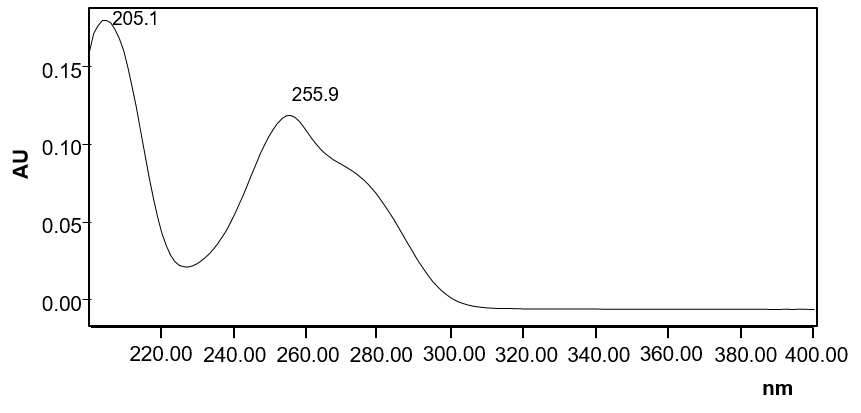


**Supplementary Figure 14.** ^1^H NMR (600 MHz) spectrum of **3** in D_2_O.

**3**

**Supplementary Figure 15.** ^13^C NMR (150 MHz, D_2_O) and DEPT spectra of compound of **3**.


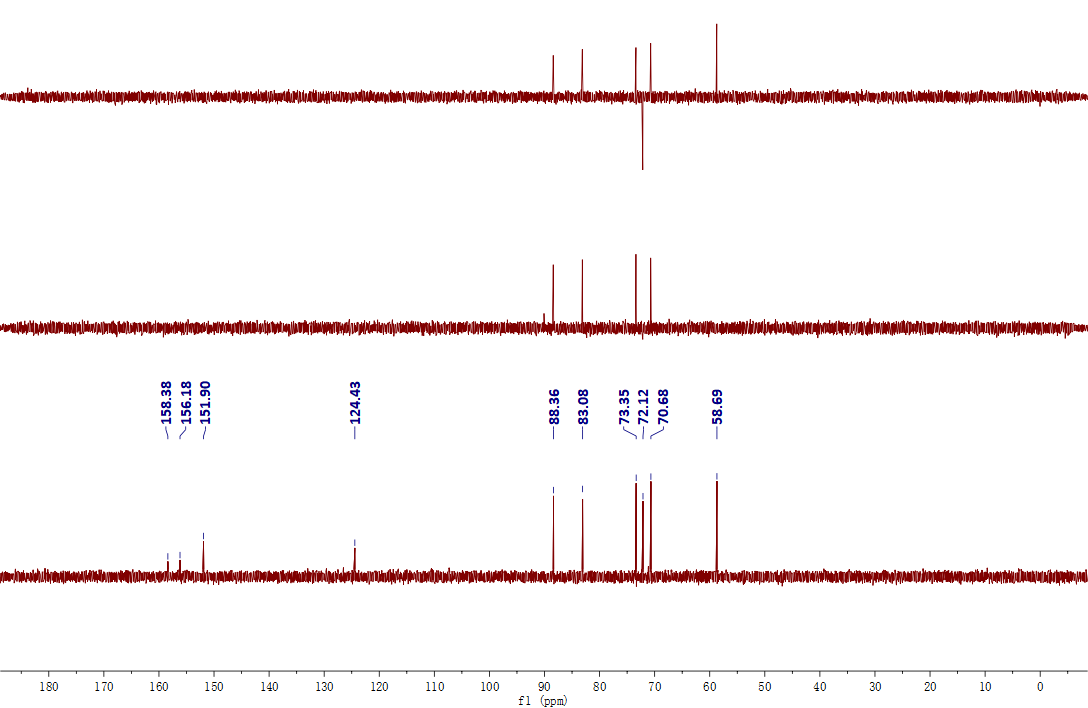


**3**

** Supplementary Figure 16.** ^1^H-^1^H COSY spectrum of **3** in D_2_O.


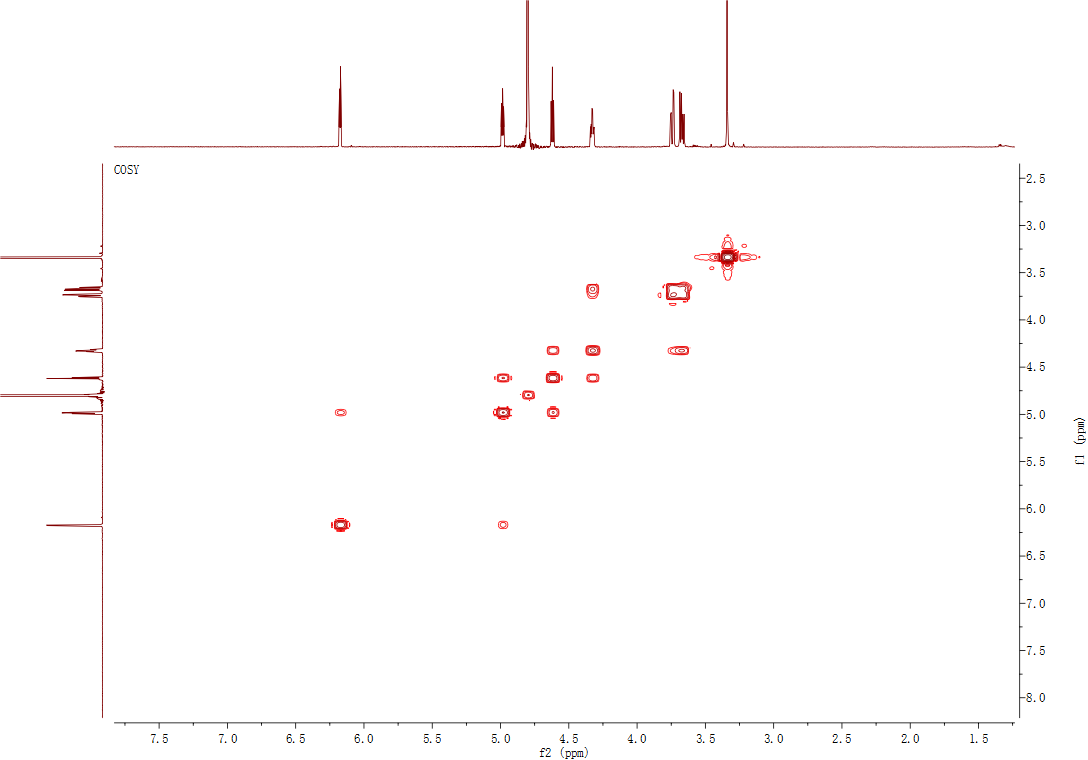


** Supplementary Figure 17.** HSQC spectrum of **3** in D_2_O

**Supplementary Figure 18.** HMBC spectrum of **3** in D_2_O.

**3**

**Supplementary Figure 19.** LC-HRMS analysis of **8-AZG-3** from mutant strain Δ8-*azgD*.

**
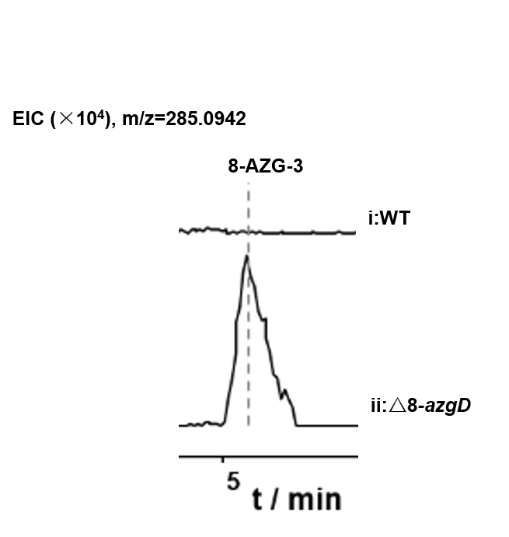
**

i): WT, *Streptomyces albus subsp. pathocidicus* CGMCC4.1633; ii): mutant strain, Δ8-*azgD*.

**Supplementary Figure 20.** HRMS spectrum of **8-AZG-3**.

**
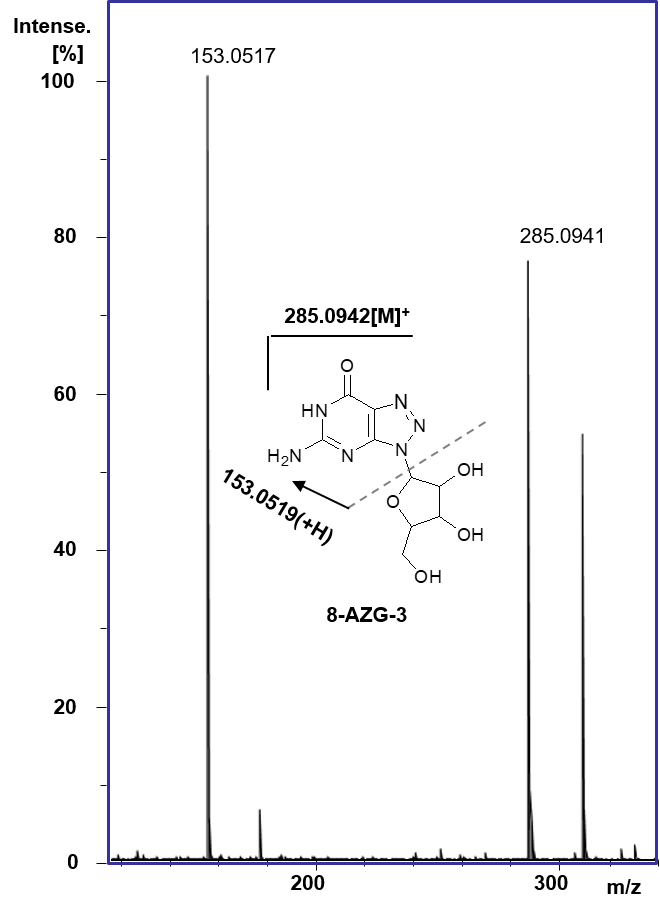
**

| Formula (M) | Ion Formula | m/z | Calc m/z | Diff (ppm) |
| --- | --- | --- | --- | --- |
| C_9_H_12_N_6_O_5_ | C_9_H_13_N_6_O_5_ | 285.0941 | 285.0942 | 0.35 |

**Supplementary Figure 21.** Sequence alignment of 8-AzgG

**
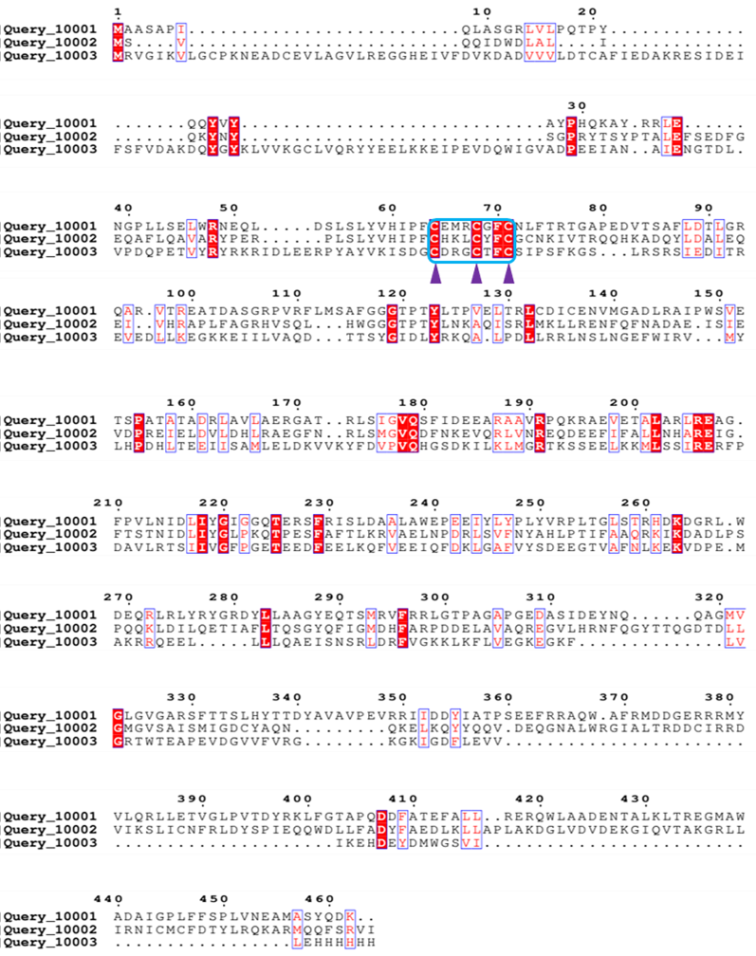
**

Sequence alignment o 8-AzgG (Query_10001) with radical-SAM enzyme HemN (Query_10002, PDB: 1OLT) from *Escherichia coli* and radical-SAM methylthiotransferases RimO (Query_10003, PDB: 4JC0) from *Thermotoga maritima*. Conserved active site residues CxxxCxxC are indicated with purple triangles.

**Reference**

(1) Nishihara, K., et al. Appl. Environ. Microbiol. 1998, 64: 1694-1699.

(2) He, Y.; Wang, Z.; Bai, L.; Liang, J.; Zhou, X.; Deng, Z., J Microbiol Biotechnol 2010, 20, 678-682

**Supplementary notes** **Streptomyces pathocidini strain CGMCC4.1633 8-azg biosynthetic gene cluster, complete sequence.**

GenBank flat file:

LOCUS MT543149 11423 bp DNA linear BCT 23-JUL-2020

DEFINITION Streptomyces pathocidini strain CGMCC4.1633 8-azg biosynthetic gene

cluster, complete sequence.

ACCESSION MT543149

VERSION MT543149

KEYWORDS .

SOURCE Streptomyces pathocidini

ORGANISM Streptomyces pathocidini

Bacteria; Actinobacteria; Streptomycetales; Streptomycetaceae;

Streptomyces.

REFERENCE 1 (bases 1 to 11423)

AUTHORS Huang,W.

TITLE Direct Submission

JOURNAL Submitted (28-MAY-2020) Biobased Materials and Chemicals, Qingdao

Institute of Bioenergy and Bioprocess Technology, Chinese Academy

of Sciences, 189 Songling Road, Qingdao, Shandong 266101, China

COMMENT ##Assembly-Data-START##

Assembly Method :: Lasergeneversion v. 7.0

Sequencing Technology :: PacBio

##Assembly-Data-END##

FEATURES Location/Qualifiers

source 1..11423

/organism="Streptomyces pathocidini"

/mol_type="genomic DNA"

/strain="CGMCC4.1633"

/isolation_source="soil"

/db_xref="taxon:1650571"

/country="China"

misc_feature 1..11423

/note="8-azg biosynthetic gene cluster"

CDS complement(1..1173)

/codon_start=1

/transl_table=11

/product="8-AzgA"

/protein_id="QLM04708"

/translation="MSPEEERCHSDTASAGPEAVRALAEEFVTLYHRERPEAGSADRR

LAEVAAEISRTGTYTHTAQELDFGAKVAWRNSIRCIGRLYWQSLIVRDRRKVDSADQI

SREIVRHLCQATNGGRIRPCVTVFAPELPRRPAPRIWNDQLVRYAGYTRSDGSILGDP

VSVDITAAAQNLGWPGGPGTPFDVLPLIVHGRDDKPRWFDLPDSAVLQVPIEHPEYEW

FARLGVRWYAVPVISGMRLEIGGVRYPLAPFNGWYMGTEIGARNLADTDRYNLLPDIA

RRLGLDTSHDRSLWKDRALVELNLAVLHSFEKAGVTISDHHTESRHFLTHIAREERAG

RTVGADWSWIVPPVSGAATPVFHRYYDTEQRYPAFVDQPKAPADSGPAARPPGRDF"

CDS complement(1170..2396)

/note="MFS transporter"

/codon_start=1

/transl_table=11

/product="8-AzgB"

/protein_id="QLM04709"

/translation="MTTPLTPHPTRQRPAPVLGRGVVLLLTVGTGLSVANNYYVQPLL

GDIGRDLGLPEGQAGLLVTVGQSAYAVALLLVLPLGDLLERRRLIVILSSACAVSLVL

MASARSEPALFLGAGLVGLCSVTTQVMVPYAASLSAPDQRGRVVGTVMAGLLCGILFA

RTVAGALAQWAGWHAVYWCAAVMMACLAATAAVFLPRYQERGAPGYRHLVASVFHLFA

REPVLRLRAVYGALSFGAFSVLWTALTFLLSAPPYGYSPGVIGLFGLFGLAGALAAAR

AGRLADRGLAHKVTGVSSVLLLAVWIPLGLGSALLPALALGIVVLDLAAQALHITNQS

EIYRLAPEIRSRLTAAYMTTYFLAGAAGSALASLAYARLGWTGVSVLGAALGAATTSL

WAISALRQRSRHRRTP"

CDS complement(2417..3127)

/note="ADP-ribose pyrophosphatase"

/codon_start=1

/transl_table=11

/product="8-AzgC"

/protein_id="QLM04710"

/translation="MLAHDGDNWHILLVRRGKEPFQGRWALPGGKVGADEDATTAARR

ELQEETGLRATDTELIPLSWRTSPDRDPRGRYVSLVYAALLPQPQQVHGGGDAAAAWW

QPLPVPPRGFPDPAAEQHFAFDHAAILRELLPAAPHGRSAVPHIREATAADGQEIRRL

LKSHALGDGAAGHPATGGLGPRTHVAALGSFLVGCAGLPPNGDPAAKDRILVHPRWQG

RGIEQELAAVQAQEHRPP"

CDS complement(3192..3695)

/note="nucleotide monophosphate nucleosidase"

/codon_start=1

/transl_table=11

/product="8-AzgD"

/protein_id="QLM04718"

/translation="MEKWPDILIAVVGKGRNCPPPVYQLAHEVGGTVARLPDCLLVTG

GLGGAMEAAARGAKENGGRVLSLLPGVSNRTTQPHTEVDIRLDTGLTANGRNIVLASV

VNAMIAVPGSHGALQEMIVAVDAGKTVWAVGEHTTRLPGVEYLASSGELSARLHAFTA

ASTSDLG"

CDS complement(3702..4010)

/note="hypothetical protein"

/codon_start=1

/transl_table=11

/product="8-AzgE"

/protein_id="QLM04711"

/translation="MADEDVGRLADQMRQALHTCSVHTVGPLAAYERCTAALDQGRID

AQQFQEIGRRLVAISEERMHAYAAQGEHPDNSPKIRREKRLIGFVDALFALGLRKKKN

"

CDS complement(4047..4667)

/note="GTP cyclohydrolase I"

/codon_start=1

/transl_table=11

/product="8-AzgF"

/protein_id="QLM04712"

/translation="MAESQQPHLPLPPKPVDVDVIENAISELLRGLGQGDKSEVMSQT

PRRVAELYAQSINPGDIDIEEDFKVFDNPGMQDLILVNDVHYVSLCEHHLAPAFGVAH

VGYVPDRKVAGYSKLKKGLNYLARQPQLNERLVVDAVNFLEARLQPKGIAMVLRSAHC

CIALRTNAPSQEVVTVIERRGALCEERYWSPLWASAVAEKPAFLGR"

CDS complement(4903..6291)

/note="Oxygen-independent coproporphyrinogen-III

oxidase-like protein"

/codon_start=1

/transl_table=11

/product="8-AzgG"

/protein_id="QLM04713"

/translation="MAASAPIQLASGRLVLPQTPYQQYVYAYPHQKAYRRLENGPLLS

ELWRNEQLDSLSLYVHIPFCEMRCGFCNLFTRTGAPEDVTSAFLDTLGRQARVTREAT

DASGRPVRFLMSAFGGGTPTYLTPVELTRLCDICENVMGADLRAIPWSVETSPATATA

DRLAVLAERGATRLSIGVQSFIDEEARAAVRPQKRAEVETALARLREAGFPVLNIDLI

YGIGGQTERSFRISLDAALAWEPEEIYLYPLYVRPLTGLSTRHDKDGRLWDEQRLRLY

RYGRDYLLAAGYEQTSMRVFRRLGTPAGAPGEDASIDEYNQQAGMVGLGVGARSFTTS

LHYTTDYAVAVPEVRRIIDDYIATPSEEFRRAQWAFRMDDGERRRMYVLQRLLETVGL

PVTDYRKLFGTAPQDDFATEFALLRERQWLAADENTALKLTREGMAWADAIGPLFFSP

LVNEAMASYQDK"

CDS 6461..7597

/note="DUF1152 domain-containing protein"

/codon_start=1

/transl_table=11

/product="8-AzgH"

/protein_id="QLM04714"

/translation="MTAKLLIAAGGGGDAITTAMVHAALHGDDAPALILTYAWERLVV

DPVPGPRRRTDFTGTERVGDQVHLITPGSAPIPPAGSLLPRLAADLRPALGLIDPYEG

TVGLALQIAQAAEYCGADRVEIVDVGGDVAADGSEPTLRSPLGDALVVAACLRTGIPA

AVHIAGPGLDSEVPEAVLLPRLGLPEFTLTAAHTNCVGNVFDWHPSEASALLVAAARG

IRGVCGTRDAPDPVVLSPASAHVYRLPLARVLEINRLAQALDGTADLAEAEEASRSVC

GFSEVARERERARQRCAARGSTRREGGAPDAASPYQRFVEWEQRTAATGIQYVTTRRF

AEELGLSQGALTALRNQLKNTAPARSAPPLWQLHSDGQLTREKS"

CDS 7863..9911

/note="hypothetical protein"

/codon_start=1

/transl_table=11

/product="8-AzgI"

/protein_id="QLM04715"

/translation="MNSAREAARNQGIDPANYDYLFVDITKHTGCNWGAQGSMPGNWI

VSNAEGHKSWMWMHEFGHNLGFNHPLTLRGCPVSGDVTRINGSCTQGATDDPGDPVGG

GGKRLYPASYRQYAGWLSGSQMIPIRTSGSYQLGVLGKDGVQEYRIDRGNGSTLSLEF

RRPKPPYDDFASDDPLVNGVTVRIVSGDGRHNTLVDATPATSSTKDAPLAAGKILVDE

VAKAAVRVCSVGDNGADLRVAIGDTSPADCKDTEELTAPTVAKAEITGVRQSTGEVVR

DISGTGVPGAIVQFLRNDGRWENNMATVEADGTWSRINTVYGADDGVTVRQTKDGETS

DASPKKSYTIAKPTIALAEIRVNGANSELSYHAAGKGVPGARIQVANASGGWTDRNVV

GGDGSWSFSGGYGNAFARVRQVLDGITSAASDDGEWKVPTPTISSAQITGVDGSGKTQ

YALEGTGLAHTTVQIRNGSSWVDSVKVGQDGKWSVAKSTATQDTSGAVVRVAGSIGGV

THYSAASAKKSYTIAKPTITSPGDGATVQASFTAHGKGVSGAQVSAKIEGVTKSVTVN

ANGDWTVDFTGVAGGKQELSVSQSRDGLVSPAERRNITVAGSAPGTPPSSCGGAPAWD

SSKIYWGGDLVFDVTDVNNPSAGYTVYRAAWWNVNVRPVTNNGPWGKEWILVGTCQAP

"

CDS 10045..10356

/note="hypothetical protein"

/codon_start=1

/transl_table=11

/product="8-AzgJ"

/protein_id="QLM04716"

/translation="MVVCGPARIPLEEINDLRRRMMGVATQESSNVPRFSSLGVGGSR

VDHTMLLCMPEKTSPESSLHGSADLSVVPVSPGVDDWIPSADELVQAIVEVMNQRCRT

N"

CDS 10590..11423

/note="glycosyltransferase"

/codon_start=1

/transl_table=11

/product="8-AzgK"

/protein_id="QLM04717"

/translation="MPASLPLHPASGTRAQSAAVIDSSAPRVLIAVDAYPSDSREAAH

FTRQLISELAAKGHEVHLVCPSDRGRPRVERVDGVIVYRLRSLPALMQPTRRVVMRLG

LRTCVERLIDRIAPDVLHVQSHSVIGRAVAGAARRAGLPVVADTSHRLLQRVRAGQGL

RLDAKPVTRGVDPAHVRPSGTLTEGTRARFRLPCRDKVPGARRQSSSWARLSVAGLGL

ALAMLATVHVPGNRDPIRDVISYYAFDSATVWPYYSWRAVACDRHVRLGHRPPPAAAA

G"

ORIGIN

1 tcaaaagtcg cgcccaggcg ggcgcgccgc aggccctgag tcggccggtg ccttcggttg

61 gtcgacaaac gccgggtagc gctgctcggt gtcgtagtag cggtggaaca cgggggttgc

121 ggcgccggag acgggtggca cgatccacga ccagtcggcg ccgacggtcc gcccggctcg

181 ttcctcgcga gcgatgtgcg tcaggaagtg gcgcgattcg gtgtggtgat cgctgatggt

241 gactcctgcc ttctcaaagg agtggagcac ggcgaggttc agctccacca gtgcgcggtc

301 cttccacaac gagcggtcgt gggaagtgtc caggcccagt cggcgggcga tatcgggcag

361 caggttgtag cggtcggtgt cagccaggtt gcgggcaccg atctccgtgc ccatgtacca

421 gccgttgaaa ggggcgagcg ggtagcgtac gccgccgatt tccagtcgca tcccggagat

481 gaccggcacc gcgtaccagc gcacacccag ccgggcgaac cactcgtact cggggtgttc

541 gatcggaacc tggagcacgg cggagtccgg caggtcgaac caccgcggct tgtcgtcgcg

601 cccgtgcacg atgagaggca ggacatcgaa gggagtgccc gggccgccgg gccagcccag

661 attctgcgcg gcggccgtga tatcgacgct gaccgggtcg ccgaggatcg aaccgtcgga

721 gcgtgtgtag cccgcgtagc gcacgagctg gtcgttccat atccgcgggg ccggccgccg

781 ggggagctcc ggggcgaaga cggtgacgca cggacgtatt cgtccgccgt tggtggcctg

841 gcacaggtgc cggacgattt cccgagagat ctggtcggcg ctgtccacct tccgccggtc

901 gcgcacgatc agcgactgcc agtacagacg gccgatgcag cggatgctgt tgcgccaggc

961 gaccttggcg ccgaagtcca gttcctgcgc cgtgtgcgtg tacgtcccgg tgcgcgagat

1021 ctctgcggcc acctcggcca gtctccggtc cgcggaaccg gcctcgggac gctcgcggtg

1081 atacaaggtc acgaactcct ccgccagcgc ccggaccgct tccggtcctg cggaggcggt

1141 gtcggagtgg cagcgctcct cctccggact cacggggttc gcctgtgccg ggagcgttgg

1201 cggagcgccg agatcgccca cagtgaggtc gtcgccgccc cgagtgccgc cccgagcacc

1261 gagaccccgg tccagccgag ccgggcgtag gccagggagg cgagcgccga ccctgcggct

1321 ccggccagga agtacgtcgt catgtaggcc gcggtgagac gggacctgat ctccggcgcc

1381 aaacggtaga tctcgctctg gttggtgatg tgcagggctt gagccgcgag atcgagcacc

1441 acgatgccca gggccagggc cggcagcagt gcgctgccca gcccgagggg gatccacacg

1501 gcgagcagca gtacggagga gacaccggtc accttgtgtg ccagtccccg gtccgcgagg

1561 cgcccggcgc gtgccgcggc cagcgccccg gccaggccga acaggccgaa caggccgatg

1621 accccgggcg agtagccgta cggcggggcg gagagcagga aggtcagggc ggtccacagc

1681 acgctgaagg ccccgaacga cagtgccccg tacaccgccc gcagccgtag caccggttcc

1741 cgggcgaaca ggtggaacac cgaggccacg aggtgccggt agccgggagc gcccctctcc

1801 tgataccggg gcaggaagac cgccgccgtt gccgccagac aggccatcat gaccgcggcg

1861 caccagtaga cggcgtgcca cccggcccac tgcgccagcg caccggccac ggtacgggcg

1921 aacaggatgc cgcacagcag ccccgccatg acggtgccca cgacgcgccc gcgctgatca

1981 ggagcggaca gggacgcggc atagggcacc atcacctggg tggtgacgga gcacagcccc

2041 accagtcctg ctcccaggaa gagggccggc tccgatcggg cgctggccat gagcaccagg

2101 ctcacggcgc aggccgagga caggatgacg atcagccggc gccgttcgag caggtcgccg

2161 aggggaagga cgaggagcag tgccaccgcg taggcgctct gtcccaccgt gaccagcagg

2221 ccggcctgtc cttccggcag gccgaggtcg cggccgatgt cgccgagcag cggctgcacg

2281 tagtagttgt tggccacgga caacccggtg ccgacggtga gcagcaagac cacgccgcgt

2341 cccagaacgg gcgccgggcg ttgtcgcgtg gggtgtggtg tgagcggtgt ggtcatggcc

2401 gctttccttt tacgagtcac ggtgggcggt gctcctgagc ctgtacggcg gccagttcct

2461 gctcgatccc ccgcccctgc cagcgggggt ggaccaggat ccggtccttc gctgccgggt

2521 caccgttcgg cggcaggccc gcacagccca cgaggaagct ccccagtgcc gctacgtgcg

2581 tccgggggcc gaggcccccg gtggccgggt ggccggcggc gccgtcgccc agggcgtgtg

2641 acttcagcag gcgccggatc tcctggccgt ccgccgcggt cgcctccctg atgtgcggga

2701 cggccgaccg gccgtgtggt gcggcgggaa gcaactcccg gaggatggcg gcatggtcga

2761 aggcgaagtg ctgctccgct gcggggtccg ggaagccgcg cggggggacg ggcagcggct

2821 gccaccacgc cgccgctgcg tccccgccgc cgtgcacctg ctgcggctgc ggcagcagcg

2881 ccgcgtagac caggcttacg tagcgtcctc tggggtcgcg gtccggtgag gtgcgccagg

2941 acagcggaat gagttcggtg tcggtggcgc gcagcccggt ttcttcctgt agttcgcggc

3001 gggccgcagt tgtcgcgtcc tcgtcggcgc cgacctttcc gccgggaagc gcccaccggc

3061 cctggaacgg ttccttgccc ctgcgaacca gcaggatgtg ccagttgtca ccgtcgtggg

3121 ccagcatcac caggtcgacg gtcaggatga cggcgctcag cgggcactcc ttgtggttgt

3181 ccgcacgccg gctacccgag atcagaggtg gaggcggcgg tgaaagcgtg caggcgcgcg

3241 gagagttcgc ccgaggacgc gaggtactcc acccccggga ggcgggtggt gtgctcgccc

3301 actgcccata cggtcttgcc ggcgtcgacg gccacgatca tctcctggag ggctccgtgc

3361 gagcccggga cagcgatcat ggcgttgacg acgctggcca gcacgatgtt gcggccgttg

3421 gcggtcagac cggtgtccag ccggatatcc acctcggtgt gcggctgggt cgtgcggttc

3481 gagacacctg gaagcaggct cagcacgcgg cctccgttct cctttgcacc gcgagccgct

3541 gcttccatcg ctcctccgag cccaccggtc accagaaggc agtcgggcag ccgtgcgacg

3601 gtccctccca cctcgtgggc caactggtac accggtggtg ggcagttgcg tcccttgccg

3661 accacggcga tgaggatatc cggccatttc tccatggggc ctcagttctt cttcttgcgc

3721 aggccgaggg cgaagagcgc gtcgacgaag ccgatcagcc gcttctcccg cctgatcttc

3781 ggcgagttgt cggggtgctc tccctgagcg gcgtacgcgt gcatgcgttc ttcggagatg

3841 gcgaccagcc tgcggccgat ctcctggaac tgctgcgcgt cgatgcgccc ttggtcgagg

3901 gcggcagtgc atctctcgta cgccgccaag gggcccacgg tatggacaga acaggtgtgc

3961 agtgcctgtc gcatctggtc ggcgagccgg cccacgtctt cgtcggccac ggacgccgac

4021 ggtgctcctc ggtccattcg gctcgcctat ctccccagga aggccggttt ctccgcgacg

4081 gcactggccc acagcggcga ccagtagcgc tcctcgcaca gtgcgccgcg ccgctcgatc

4141 accgtgacga cttcctggct cggggcattg gtgcgcagcg cgatgcagca gtgcgcactg

4201 cgcaggacca tggcgattcc cttcggctgc agccgcgcct ccaggaagtt caccgcatcg

4261 accaccaggc gctcgttgag ctgaggctga cgcgcgaggt agttcagccc cttcttgagc

4321 ttggagtagc cggccacttt gcggtcggga acatatccga catgggcgac cccgaaggcg

4381 ggcgccagat ggtgttcgca cagcgagacg tagtgcacgt cgttgaccag gatcaggtcc

4441 tgcattccgg ggttgtcgaa gaccttgaag tcctcttcga tgtcgatgtc gcccggattg

4501 atcgactggg catacaactc cgccacccgg cgcggagtct gcgacatcac ctcgctcttg

4561 tcaccctggc cgagaccgcg cagcagttcg ctgatggcgt tctcgatcac gtccacgtcc

4621 accggcttgg ggggaagcgg taaatgaggt tgctgggatt cagccatggt gtcatcctcc

4681 aggtctttgg gcccgctttg tgggcggaac tctccaggac gcgtctttta gagtctgcac

4741 ggaacgatcg tttggcagag gctagaaacc ggatgggagc gagtcaaccg gtgagcagcg

4801 aaaacacgcc aggtgttcgt gtggtgcccg cgcacggaaa ttccccgatg gcacaggcca

4861 tttcgtggcc tctccgtgcg cggggcaccg gggttcgctg cgctacttgt cctggtagct

4921 cgccatggct tcgttgacca ggggggagaa gaagagcggg ccgatcgcat ccgcccaggc

4981 catcccctcc cgggtgagtt tcagcgcggt gttctcgtcg gcggcgagcc actgcctttc

5041 gcggagcagg gcgaactctg tcgcgaagtc gtcctggggc gccgtaccga acagcttccg

5101 gtagtcggtg accggcaggc cgacggtctc cagcagccgc tgcaggacat acatccggcg

5161 ccgttccccg tcgtccatcc ggaaggccca ctgggcccgg cggaactctt cggagggggt

5221 ggcgatgtag tcgtcgatga tgcgccgcac ttcgggcacg gccaccgcgt aatcggtggt

5281 gtagtgcagg gaagtggtga acgaacgggc gccgacgccg agtcccacca tgccggcctg

5341 ctggttgtac tcgtcgatgc tcgcgtcttc ccccggggcg ccggcgggtg tccccagccg

5401 tcggaagacc cgcatcgagg tctgctcgta gccggcggcc agcaggtagt cgcggccgta

5461 ccggtacagg cgcaggcgtt gctcgtccca cagcctgccg tccttgtcgt gccgggtgct

5521 cagaccggtc aaggggcgta cgtagagcgg gtacaggtag atctcctccg gctcccaggc

5581 cagcgcggca tcgagggaga tacggaagga gcgctctgtc tggccgccga tgccgtagat

5641 gaggtcgatg ttcaagacgg ggaagccggc ctcgcgcagc cgggccaggg ccgtctccac

5701 ctccgcgcgt ttctgcggtc gtacggcggc gcgggcttcc tcgtcgatga agctctgtac

5761 accgatgctc agccgggtgg cgccgcgctc ggcgagcacc gcgagccggt cggcggtcgc

5821 ggtggccgga gaggtctcca ccgaccaggg gatggctctg aggtctgccc ccatcacgtt

5881 ctcgcagatg tcgcagagcc gggtgagctc tacgggggtg agataggtgg gggtgccgcc

5941 gccgaaggcg gacatcagga agcgaaccgg ccgcccgctt gcgtctgtcg cttcacgggt

6001 cacccgcgct tgccgtccca gcgtgtccag aaacgcgctg gtgacgtctt ccggggcgcc

6061 ggtacgggtg aacagattgc agaatccgca gcgcatctcg cagaacggta tgtgcacgta

6121 gagcgagagg gagtcaagct gctcgttcct ccacaactcg ctcagtaacg gcccgttctc

6181 cagccgccgg taggcttttt gatggggata ggcgtagacg tactgctggt agggagtctg

6241 cggcaaaact aatctgcccg aggccagttg gatgggggca gaggcggcca tggacaacac

6301 cttccctctc ttgacaaggg agctgaagta cgactctgcg gacacggtag cggtcaggta

6361 cacgcatacg taagcatggc gggccacctc tcggagtggc ccgggcattt tcggtcagtg

6421 gacggacgga caccgggaac gacccagggg gtcaaggcat ttgactgcga aacttctgat

6481 tgccgccgga ggcggcgggg acgcgatcac caccgcgatg gtccacgcgg ctctgcacgg

6541 tgacgacgct cccgccctca tcctgaccta tgcctgggaa cgcctggtgg tcgatccggt

6601 acccggcccc agacgccgca cggacttcac cggcaccgaa cgcgtcggtg accaggtgca

6661 cctgatcacg cccgggtcgg cccccatccc tcccgcgggc tcactgctgc ctcggctcgc

6721 ggccgacctg cggcccgcgc tcggcctcat cgacccgtac gagggcacgg tgggcctggc

6781 cctccagatc gcccaagccg ccgaatactg cggcgccgac cgcgtcgaaa tcgtcgacgt

6841 cggcggggac gtggcggccg acggcagcga acccaccctg cgcagcccgc tgggcgacgc

6901 gctcgtcgtg gcggcgtgcc tgcgcaccgg tataccggcc gcggtgcaca tcgccggccc

6961 cggactcgac tccgaggtgc ccgaagccgt actgctgccc cgcctgggcc tcccggagtt

7021 caccctcacc gccgcacaca cgaactgcgt cggcaacgtc ttcgactggc acccctccga

7081 ggcgagcgcc ctcctggtgg ccgcggcccg cggcatacgg ggtgtctgcg gtacccgcga

7141 cgcgccggac ccggtcgtgc tcagccccgc cagcgcacac gtctaccggc tccccttggc

7201 ccgtgtcctg gagatcaacc ggctggccca ggcgctcgac ggcactgccg acctggcgga

7261 agccgaggaa gccagccgct cggtgtgcgg tttctccgag gtcgcccgcg agcgcgagcg

7321 cgcccgtcag cggtgtgcgg cgcgaggcag tacgcggcga gagggcggtg caccggacgc

7381 cgcctccccc taccagcggt tcgtcgagtg ggagcagcgc accgcggcca ccggcatcca

7441 gtacgtcacc acccgccggt tcgccgagga actgggcctc tcccagggcg cactgacggc

7501 cctccgcaac cagctgaaga acaccgctcc cgcgcgctcc gcaccacctc tgtggcagct

7561 gcactccgat ggacagctca cccgggagaa gagctgaagc cggcgacgcc cacaggcgca

7621 agatccccga gaagtccaac gaatgccgcc gcaccttccc tgacccgctc gactcgtaac

7681 tttagtcagc ccagttcctt acttcctgca tggctcgtgg cggaaacgac ctcttaggct

7741 tgtggggatc ccggtctgac gcagagaatc aacctgccat ttcgcgagaa gtcctccgcg

7801 tctccagagc tcgtttcgaa aggctcggcg ccaggccgag cgactgcaaa tcggattcga

7861 tattgaattc agcgcgagaa gcggcgcgaa atcaggggat agaccccgcg aactacgact

7921 acctttttgt tgatatcacg aagcacaccg gatgcaattg gggtgcccag ggttccatgc

7981 ccggaaactg gatcgtgtcc aacgccgagg gccacaagtc gtggatgtgg atgcacgagt

8041 tcggacacaa tctcggattc aaccaccctc ttacgctacg gggctgccct gtgtcggggg

8101 acgtgacgcg gatcaacggc agctgtaccc aaggagcgac tgacgatccg ggagaccccg

8161 tgggcggtgg cggcaagcga ctctacccgg ccagttaccg gcagtacgcg ggctggctct

8221 ccggttccca gatgatcccg atccgcacca gcgggtcgta ccagctgggc gtcctgggga

8281 aggacggtgt ccaggagtac cggatcgacc ggggcaacgg ctccactctg tcactggaat

8341 tccgtcgccc caagccgccc tatgacgact ttgcctcgga cgacccgctg gtgaacggcg

8401 tcaccgtgcg tatcgtcagc ggggacggca ggcacaacac gctcgtcgac gccactcccg

8461 caacgtcgag caccaaggac gcccccctgg cggcgggcaa gatcctggtg gacgaggtcg

8521 ccaaggcggc cgtccgggtc tgctcggtgg gcgacaacgg cgctgacctc agggttgcca

8581 tcggtgacac ctcgcccgcc gactgcaaag acacggagga gctgacggct ccgaccgtgg

8641 ccaaggccga gatcaccggg gtccgtcagt ccacgggtga ggtggtccgc gacatttccg

8701 gcactggtgt gccgggggcg atcgtgcagt tcctgcggaa cgatggcagg tgggagaaca

8761 acatggccac ggtcgaggcc gatggcacct ggtcgcggat caacactgtc tacggtgccg

8821 atgatggcgt gacggttcgg cagaccaagg atggtgagac gtccgacgcc tccccgaaga

8881 agtcctacac gattgccaag ccgacgatcg ccctggcgga aatccgagtg aatggtgcga

8941 actccgagct ctcctatcac gctgccggca agggagtgcc aggtgctcga atccaggtag

9001 cgaacgcgtc cggcggctgg acagaccgca acgttgtcgg cggagacggc tcctggtcat

9061 tctctggcgg gtacggtaac gcttttgcac gcgtccggca ggtgctcgac ggcatcacat

9121 cagccgcttc ggatgatggg gagtggaagg tcccgacccc gacgatctcc tccgctcaga

9181 tcacgggcgt ggacgggagc ggcaagacgc agtacgcgct ggaaggcacc ggtctggcac

9241 ataccacggt gcagatcagg aatggttctt cctgggtgga ctcggtgaag gtgggtcagg

9301 acggcaagtg gtcggtagcc aagtccaccg cgacgcaaga cacctccggt gcggtggtgc

9361 gtgtcgcggg cagcatcggc ggcgtcaccc actactcggc ggcttccgcg aagaagtcct

9421 acacgattgc caagccaacc attacctcgc ccggtgatgg agccacggtg caggcctcgt

9481 tcaccgcaca tggcaagggc gtatccggtg cccaggtctc ggccaagatc gaaggcgtga

9541 ccaagagcgt gaccgtgaac gcgaacggcg actggacagt cgacttcact ggcgtggccg

9601 ggggcaagca ggagctgtcc gtctcccagt cgcgtgatgg cctggtctcc ccggcagagc

9661 ggcgcaacat cacggttgcg ggctcggccc cggggacgcc gccctcctct tgtgggggtg

9721 ccccggcctg ggattcctcc aagatttact ggggcggtga cctcgtgttc gacgtgaccg

9781 acgtcaacaa tccgtcggcg ggatacaccg tctaccgggc tgcttggtgg aacgtgaatg

9841 tccgcccagt gaccaacaac ggcccctggg gcaaggagtg gatcctggta ggcacctgcc

9901 aggctccctg acgcgagaac gctccggccc tcactggagc ccgtgactgc agtccagtca

9961 cgggctccgg tgcgcctcac tcggcgtggg tgcgggcgcg ggtcgtcgaa caggtcttgc

10021 ccgaggcggg atcggagctt gtgggtggtg gtgtgcggtc cggctcggat acccctggaa

10081 gagatcaacg acctgcgtcg tcgaatgatg ggagtggcca ctcaagagtc ttccaacgtc

10141 cctcgatttt catctttggg cgtcggaggg agccgagtcg atcacaccat gctcttgtgc

10201 atgcccgaga aaacctcgcc cgagtcaagc ctgcacggtt ccgctgatct atccgtcgtg

10261 ccggtgagcc ctggcgtcga tgattggatt ccgtcggccg acgaactcgt ccaggccatc

10321 gtggaggtca tgaaccagcg gtgccggacg aattgaacag ccatcattcg tcgtttgccg

10381 aatatccggt gtgagaccgc cgggcagacg tccgccgttg catgagccgc gttcgtcgcc

10441 cccatggcgc ctaccacaga tggttgcagc aagcgggcac ggcatgagct tgctcgcctg

10501 acttctcttg cagtcgtcca ggttcccggg cgcgcagtcc accggagagc tcatcagagt

10561 ccgacgccgc tgcaggcaag aggagtccca tgcccgcgtc ccttccgctg caccctgcct

10621 ccggcacccg cgcacagtct gccgcggtga tcgacagctc ggctccccgc gtactgatcg

10681 ccgtcgacgc ctatccgtcg gactcccgcg aggccgccca tttcactcgc caactgatct

10741 ccgaactggc ggcgaagggg catgaggtgc atctcgtgtg cccgtcggat cggggccgtc

10801 cgagggtgga gcgagtggac ggcgtcatcg tctaccgtct gcgctccctg cctgcgctga

10861 tgcagccaac acgacgagtg gtcatgcgtc ttgggctgcg tacctgtgtc gagcggctga

10921 tcgaccggat tgctcccgat gtgctgcatg tgcagagcca ctccgtgatc ggccgggccg

10981 tggccggcgc cgcacggcgt gcggggctgc ccgtggtggc ggacaccagc catcgcctgt

11041 tgcagcgggt gcgggcagga caagggctcc gcctcgacgc caagcccgtc acccgcggcg

11101 tcgacccggc tcacgtccgc ccttctggca cactcacgga gggaactcgc gcccgtttcc

11161 gcctgccctg tcgggacaag gtgccggggg cgcggcggca gtcgtcgtca tgggccaggc

11221 tctccgtcgc cgggctcggg ttggccctgg ccatgctggc caccgtgcac gtgccgggca

11281 accgggaccc gatccgtgac gtcatcagct actacgcctt cgactcggcg accgtctggc

11341 cgtactacag ttggcgggct gtcgcttgcg accggcacgt tcgccttggg catcgccctc

11401 caccggctgc tgccgcgggg tag

//
